# Anti-Hyperglycemic and Antioxidant Effects of *Sclerocarya birrea* Leaf Crude Extract and Biosynthesized Silver Nanoparticles In Vitro

**DOI:** 10.3390/ijms27062584

**Published:** 2026-03-11

**Authors:** Sphamandla Hlatshwayo, Yamkela Ngxata, Mandisa Mathenjwa, Nokukhanya Thembane, Siboniso Percival Sithole, Sanele Nobleman Mhlungu, Bhekumuzi Prince Gumbi, Suresh Babu Naidu Krishna, Nceba Gqaleni, Mlungisi Ngcobo

**Affiliations:** 1 Sub-Discipline of Traditional Medicine, University of KwaZulu Natal, Durban 4041, South Africa; 210505551@stu.ukzn.ac.za (S.H.); 218000022@stu.ukzn.ac.za (Y.N.); thembane@mut.ac.za (N.T.); 211513812@stu.ukzn.ac.za (S.P.S.); 209504114@stu.ukzn.ac.za (S.N.M.); gqalenin@ukzn.ac.za (N.G.); 2 School of Chemistry and Physics, University of KwaZulu Natal, Durban 4000, South Africa; 219004850@stu.ukzn.ac.za (M.M.); gumbib@ukzn.ac.za (B.P.G.); 3 Department of Biomedical Sciences, Mangosuthu University of Technology, Durban 4026, South Africa; 4 Institute for Water and Wastewater Technology, Durban University of Technology, Durban 4000, South Africa; sureshk@dut.ac.za; 5 Africa Health Research Institute, Durban 4013, South Africa

**Keywords:** diabetes mellitus, *Sclerocarya birrea* leaves, silver nanoparticles, carbohydrate digestive enzymes, DPP-IV inhibition, antioxidant activity

## Abstract

Postprandial hyperglycemia represents a critical therapeutic target in type 2 diabetes mellitus (T2DM), requiring interventions that simultaneously address glycemic dysregulation and oxidative stress. This study evaluated the anti-hyperglycemic and antioxidant properties of *Sclerocarya birrea* leaf crude extract (CE) and biosynthesized silver nanoparticles (AgNPs). Phytochemical screening, nanoparticle characterization (UV–Vis, XRD, TEM, SEM, DLS, FTIR), enzyme inhibition assays (α-amylase, α-glucosidase, DPP-IV), glucose dynamics in Caco-2 cells, and antioxidant assays (DPPH, total antioxidant capacity, H_2_O_2_ cytoprotection) were performed. Phytochemical analysis identified flavonoids, tannins, alkaloids, and terpenoids as major constituents of *Sclerocarya birrea* leaf extract. AgNPs exhibited spherical morphology (36.8 ± 8.6 nm, n = 100 particles analyzed), face-centered cubic crystallinity (crystallite size: 32.1 nm), and characteristic surface plasmon resonance at 451 nm. Both formulations inhibited α-amylase (CE IC_50_: 14 µg/mL; AgNPs IC_50_: 14.07 µg/mL, n = 3) and α-glucosidase (CE IC_50_: 15.96 µg/mL; AgNPs IC_50_: 15.82 µg/mL, n = 3), showing substantial inhibition, though less potent than acarbose. Uniquely, AgNPs demonstrated selective DPP-IV inhibition (IC_50_: 220.5 µg/mL, n = 3, *p* < 0.001 vs. CE), completely absent in CE. In antioxidant assays, DPPH scavenging potency was comparable between formulations (CE IC_50_: 23.45 µg/mL; AgNPs IC_50_: 22.26 µg/mL, n = 3), while CE achieved higher maximal scavenging at the tested concentrations. Conversely, AgNPs provided superior intracellular cytoprotection against H_2_O_2_-induced oxidative stress in kidney cells (80.2 ± 2.1% viability at 76 µg/mL vs. CE 69.8 ± 3.4% at 190 µg/mL, n = 3, *p* < 0.001), representing a 2.5-fold dose advantage. Neither formulation significantly altered glucose uptake or SGLT1 expression in intestinal epithelial cells (*p* > 0.05, two-way ANOVA, n = 3). These findings establish *S. birrea*-based formulations, particularly AgNPs, as promising multifunctional candidates for managing postprandial hyperglycemia and oxidative complications in T2DM. They also highlight nanotechnology-enhanced phytomedicine as an innovative therapeutic strategy.

## 1. Introduction

Postprandial hyperglycemia, characterized by elevated blood glucose levels following food consumption, represents a significant metabolic challenge in diabetic patients [1,2]. This condition arises from impaired regulation of glucose homeostasis, primarily involving dysregulation of key enzymes and transporters in the gastrointestinal tract, including α-amylase, α-glucosidase, sodium-dependent glucose transporter 1 (SGLT1), and dipeptidyl peptidase-IV (DPP-IV) [3,4,5,6].

Carbohydrate digestion initiates with α-amylase, an endo-enzyme that catalyzes the hydrolysis of polysaccharides into oligosaccharides and disaccharides by cleaving α-D-(1→4) glycosidic bonds [7,8]. Subsequently, α-glucosidase further breaks down these intermediates into monosaccharides, such as glucose, which are then absorbed across the small intestinal epithelium [4,9,10]. Glucose absorption into enterocytes is mediated by SGLT1 and facilitative glucose transporter 2 (GLUT2), with SGLT1 being the primary active transporter at the apical membrane [11,12].

Furthermore, insulin secretion is a critical component of postprandial glucose metabolism, which is largely regulated by glucagon-like peptide-1 (GLP-1) and DPP-IV. GLP-1 is an incretin hormone that stimulates meal-induced insulin secretion. However, its activity is susceptible to rapid degradation by the DPP-IV enzyme. Consequently, increased DPP-IV activity leads to attenuated insulin secretion, thereby disturbing glucose uptake after meals [13,14].

Research indicates that inhibition of α-amylase, α-glucosidase, SGLT1, and DPP-IV arrests the breakdown of carbohydrates into glucose, thereby prolonging carbohydrate digestion. This mechanism directly reduces the rate of glucose absorption in the small intestines and alleviates postprandial hyperglycemia [4,7,8,9,10,11,12,15]. This indirect therapeutic approach to regulating blood glucose levels is particularly pertinent, as fewer than 50% of diabetic patients achieve desired glycemic control when using conventional medicines that act directly or indirectly on insulin-sensitive tissues [16]. Therefore, exploring indirect antidiabetic pathways via carbohydrate digestive enzyme inhibition offers a promising strategy that can complement direct antidiabetic approaches to achieve optimal glucose control.

Beyond glycemic control, elevated blood glucose levels observed during postprandial glycemic episodes in diabetic patients significantly contribute to the development of oxidative stress [17]. Oxidative stress, defined as an imbalance between reactive oxygen species (ROS) production and the activation of antioxidant defense systems, leads to oxidation of cellular structures, resulting in cellular and tissue damage. This, in turn, impairs glucose metabolism and exacerbates diabetic comorbidities, including microvascular complications (retinopathy, nephropathy, and neuropathy) and macrovascular complications (ischemic heart disease, stroke, and peripheral vascular disease) [18,19,20,21,22]. Consequently, targeting glucose control is crucial for preventing hyperglycemia-induced complications [23,24,25].

Currently, clinically used medications such as acarbose, voglibose, and miglitol effectively modulate postprandial hyperglycemia in type 2 diabetic patients. However, these medications are associated with adverse side effects, including severe allergic reactions, abdominal discomfort, flatulence, diarrhea, bloating, and hepatotoxicity. Given that oral antidiabetic agents often lose their efficacy with prolonged use, polytherapy is frequently required for diabetic patients. Based on the limitations of conventional antidiabetic medications and complications arising from sustained hyperglycemia, an ideal antidiabetic drug should alleviate hyperglycemia, target diabetes mellitus (DM)-induced oxidative stress, and present minimal side effects [26].

Herbal remedies are frequently considered less harmful and associated with fewer side effects compared to synthetic medications [27,28]. *Sclerocarya birrea* (A. Rich.) Hochst. subsp. caffra (Sond.) Kokwaro (Anacardiaceae), commonly known as marula, is a medium-sized deciduous African tree with a long history of use in African traditional medicine formulations for various diseases, including DM [29,30,31,32,33,34]. Its leaves, stem bark, roots, and fruits are utilized in food, beverages, and African traditional medicine formulations [35]. While the stem bark is widely used across the African continent for DM management, its harvesting presents an environmental concern due to its destructive nature [36,37]. The seasonal availability and competition for *S. birrea* fruits also limit their consistent use [38,39,40,41,42]. Therefore, this study focuses on utilizing *S. birrea* leaves as an alternative source of bioactive phytoconstituents, given that the main bioactive compounds in leaves are often similar to those in other plant parts [43].

Scientific research supports the use of *S. birrea* formulations in treating diseases related to DM. Characterization of bioactive antidiabetic compounds reported for *S. birrea* plant extract in Mali led to the marketed medication Diabetesane [44,45]. In addition to their antidiabetic properties, *S. birrea* plant extracts possess antioxidant properties [44].

To overcome pharmacokinetic limitations associated with crude herbal extracts and traditional medicines, such as issues with efficacy, permeability, and bioavailability [46,47], this study proposes the synthesis of silver nanoparticles (AgNPs) using *S. birrea* leaf crude extract (CE). This innovative approach aims to improve drug delivery and potentially lead to the development of new drug formulations from natural resources through enhanced delivery mechanisms [48,49,50,51,52,53,54,55]. Therefore, this study evaluated the anti-hyperglycemic and antioxidant properties of *S. birrea* leaf crude extract (CE) and biosynthesized silver nanoparticles (AgNPs).

## 2. Results

### 2.1. Characterization of Biosynthesized Silver Nanoparticles

#### 2.1.1. UV–Visible Spectroscopy

The formation of AgNPs was confirmed by the appearance of a characteristic surface plasmon resonance (SPR) peak at 451 nm in the UV–Vis spectrum (Figure S1, Supplementary Materials), consistent with the optical properties of spherical silver nanoparticles [40,55].

#### 2.1.2. X-Ray Diffraction (XRD)

XRD analysis confirmed the crystalline nature of the AgNPs, showing five characteristic peaks at 2θ values of 37.93°, 44.08°, 64.10°, 76.97°, and 81.07°, corresponding to the (111), (200), (220), (311), and (222) diffraction planes of the face-centered cubic (FCC) structure of metallic silver (Table S1 and Figure S2, Supplementary Materials). The maximum intensity peak was observed at the (220) plane, indicating the preferred crystallographic orientation. The crystallite size calculated using the Debye–Scherrer equation was 32.1 nm, consistent with TEM observations. Additional minor peaks in the XRD pattern are attributed to crystallized bioorganic components from the plant extract [40,55].

#### 2.1.3. Transmission Electron Microscopy (TEM)

TEM analysis revealed that the AgNPs were predominantly spherical in shape with some agglomeration. Particle size distribution ranged from 22 to 50 nm, with an average diameter of 36.8 ± 8.6 nm (Figure S3a,b, Supplementary Materials). The close agreement between TEM-measured particle size (36.8 nm) and XRD-calculated crystallite size (32.1 nm) indicates that most particles consist of single-crystalline domains.

#### 2.1.4. Scanning Electron Microscopy (SEM)

SEM analysis confirmed the spherical morphology observed by TEM and revealed some degree of particle agglomeration (Figure S4, Supplementary Materials), consistent with the modest zeta potential values discussed below.

#### 2.1.5. Dynamic Light Scattering (DLS)

The hydrodynamic diameter measured by DLS was 220 nm with a polydispersity index (PDI) of 0.334 (Figure S5, Supplementary Materials), substantially larger than the TEM-determined core size (36.8 nm). This approximately 6-fold difference reflects multiple contributing factors: (1) the hydration shell and surface-bound phytochemicals add an estimated 10–20 nm to the particle radius, (2) the PDI value of 0.334 indicates moderate size distribution (PDI 0.1–0.4 range) suggestive of some particle agglomeration, and (3) DLS employs intensity-weighted size distribution that tends to overestimate the average size for polydisperse samples, as larger particles scatter light more intensely. The modest zeta potential value (−19.5 mV, discussed in Section 2.1.6) further supports the occurrence of partial agglomeration, consistent with observations in SEM imaging (Figure S4). Despite this agglomeration, the nanoparticles remain in the nanosize range with acceptable stability for in vitro applications.

#### 2.1.6. Zeta Potential

The zeta potential was measured at −19.5 ± 8.36 mV (Figure S6, Supplementary Materials), indicating modest colloidal stability. While this value is below the ±30 mV threshold typically considered optimal for long-term stability [39], it suggests moderate electrostatic repulsion between particles. The negative charge is attributed to bioorganic capping agents from the plant extract adsorbed onto the nanoparticle surface, including polyphenolic compounds and proteins.

#### 2.1.7. Fourier-Transform Infrared Spectroscopy (FTIR)

FTIR analysis identified functional groups involved in AgNP stabilization (Figure S7, Supplementary Materials). Characteristic peaks were observed at 3653.16 cm^−1^ (O-H stretching), 3223.91 cm^−1^ (N-H stretching), 2916.88 cm^−1^ (C-H stretching), 1604 cm^−1^ (C=O stretching), 1030.4 cm^−1^ (CO-O-CO anhydride), and 873.92 cm^−1^ (aromatic bending). The shift of N-H stretching vibrations from 3244.21 cm^−1^ in CE to 3223.91 cm^−1^ in AgNPs suggests binding of amine groups to the nanoparticle surface, consistent with phytochemical capping [15,16]. The preservation of C=O peaks at similar positions indicates that carboxylic acid-containing compounds remain largely unmodified but may participate in electrostatic stabilization through their ionized carboxylate groups.

#### 2.1.8. Physicochemical Characterisation Overview 

Key physicochemical parameters of the biosynthesized AgNPs are summarized in Table 1.

### 2.2. Qualitative Phytochemical Screening of S. birrea Leaf CE and AgNPs

Qualitative phytochemical screening of *S. birrea* leaf CE and AgNPs was performed to identify major secondary metabolite classes (Table 2). Both CE and AgNPs retained most major phytochemical classes, including flavonoids, saponins, tannins, glycosides, terpenoids, steroids, and alkaloids. However, carbohydrates were present in CE but absent in AgNPs, suggesting consumption during nanoparticle synthesis as reducing agents.

### 2.3. Cell Viability Studies

Cell viability was assessed using the CellTiter-Glo^®^ Luminescent Cell Viability Assay, which quantifies ATP as an indicator of metabolically active cells. IC_10_ values (concentrations causing 10% viability reduction) were determined for both cell lines as follows:

- Caco-2 cells: CE (IC_10_ = 164.4 µg/mL); AgNPs (IC_10_ = 250 µg/mL);
- HEK-293T cells: CE (IC_10_ = 95.03 µg/mL); AgNPs (IC_10_ = 38.45 µg/mL).

**Figure 1:**
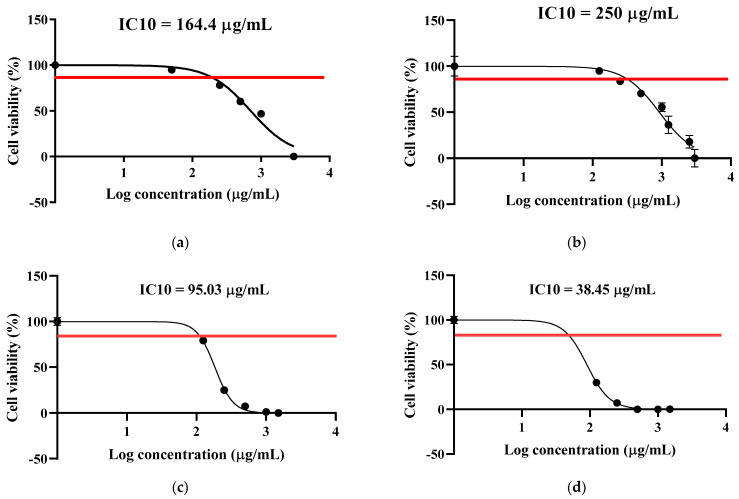
Cell viability of small intestinal (Caco-2) cells and kidney (HEK-293T) cells following treatment with various doses of *S. birrea* leaf CE and AgNPs after 24 h. (**a**) Caco-2 cells treated with CE; (**b**) Caco-2 cells treated with AgNPs; (**c**) HEK-293T cells treated with CE; (**d**) HEK-293T cells treated with AgNPs. Data presented as mean ± SD (n = 3). Statistical significance: *p* < 0.05, *p* < 0.01, *p* < 0.001 vs. untreated control (one-way ANOVA with Tukey’s post hoc test). The red horizontal line indicates the 90% viability threshold (IC10 criterion) used to guide the selection of non-cytotoxic working concentrations for subsequent assays.

AgNPs demonstrated lower cytotoxicity in intestinal cells when compared to the CE (250 vs. 164.4 µg/mL), which is relevant for oral antidiabetic applications. However, the AgNPs were significantly more cytotoxic in kidney cells than the CE (38.45 vs. 95.03 µg/mL for CE) (Figure 1).

Subsequent experiments utilized concentrations at or below 75% of IC_10_ values to minimize cytotoxic confounding effects. All results are presented with corresponding cytotoxicity data to allow assessment of potential confounding.

### 2.4. α-Amylase Activity Inhibition

Both *S. birrea* leaf crude extract (CE) and AgNPs inhibited α-amylase activity. IC_50_ values were calculated using nonlinear regression restricted to the monotonic concentration range (see Methods and Supplementary Materials), yielding IC_50_ = 14.0 µg/mL for CE and 14.07 µg/mL for AgNPs (Table 3). At the cytotoxicity-guided working concentrations used in subsequent assays, both formulations produced high levels of inhibition, consistent with strong enzymatic targeting within the non-cytotoxic exposure window. The α-amylase inhibition assay was performed using the CNPG3 chromogenic method [56].

### 2.5. α-Glucosidase Activity Inhibition

CE and AgNPs inhibited α-glucosidase activity. IC_50_ values were calculated using nonlinear regression restricted to the monotonic portion of the response curve (see Methods and Supplementary Materials), resulting in IC_50_ = 15.96 µg/mL for CE and 15.82 µg/mL for AgNPs (Table 4). These values indicate comparable inhibitory potency of the crude extract and nanoformulated preparation against α-glucosidase.

### 2.6. Extracellular Glucose Dynamics in Caco-2 Cells

**Figure 2:**
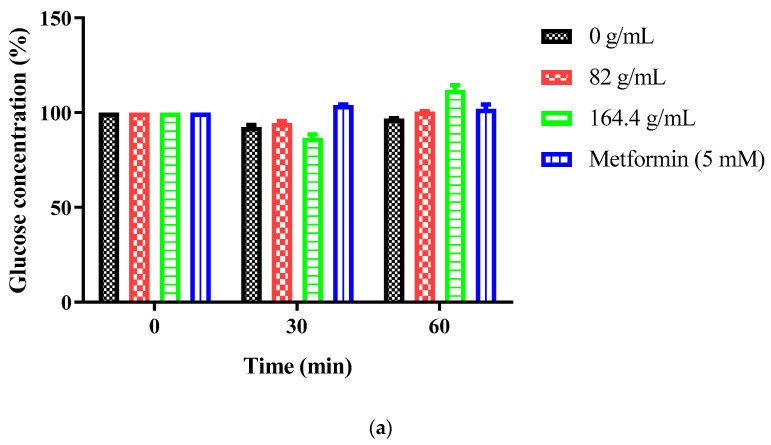

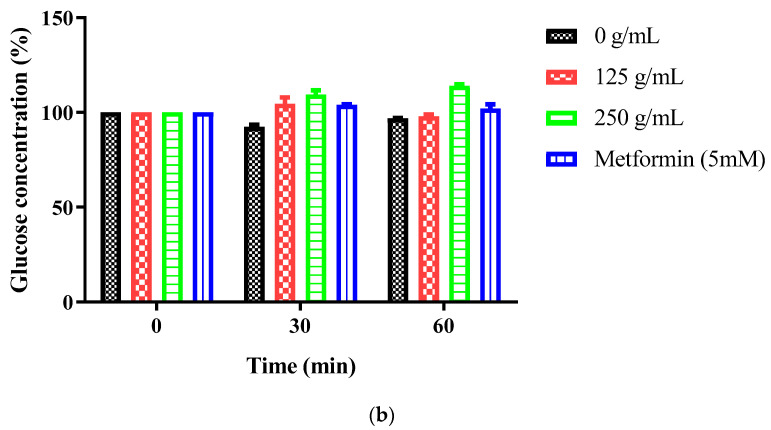
Glucose concentration in small intestinal epithelial cells following treatment with various doses of *S. birrea* leaf formulations. (**a**) Effects of CE treatment on extracellular glucose concentration over 60 min. (**b**) Effects of AgNP treatment on extracellular glucose concentration over 60 min. Data presented as mean ± SD (n = 3). No significant differences were observed between treatment groups and controls at any time point (two-way ANOVA with Bonferroni post hoc test, *p* > 0.05).

Treatment with CE at concentrations of 82 and 164 µg/mL did not significantly alter extracellular glucose concentrations at any time point examined (Figure 2a). Glucose levels remained at approximately 100% relative to baseline (time 0) at both 30 and 60 min, comparable to untreated control cells. This stability was consistent across all tested concentrations, indicating that CE neither enhanced nor inhibited measurable glucose clearance from the culture medium under these experimental conditions.

Metformin (5 mM), included as a reference compound, similarly maintained extracellular glucose at approximately 100% throughout the experimental period.

AgNP-treated cells showed transient elevation to ~110–115% at 30 min (250 µg/mL), normalizing to ~100% at 60 min (Figure 2b). No significant differences were observed (two-way ANOVA, *p* > 0.05).

### 2.7. SGLT1 Expression Analysis in Caco-2 Cells

To further investigate whether *S. birrea* formulations affect intestinal glucose transport at the molecular level, we analyzed SGLT1 expression in Caco-2 cells following treatment with CE and AgNPs.

**Figure 3:**
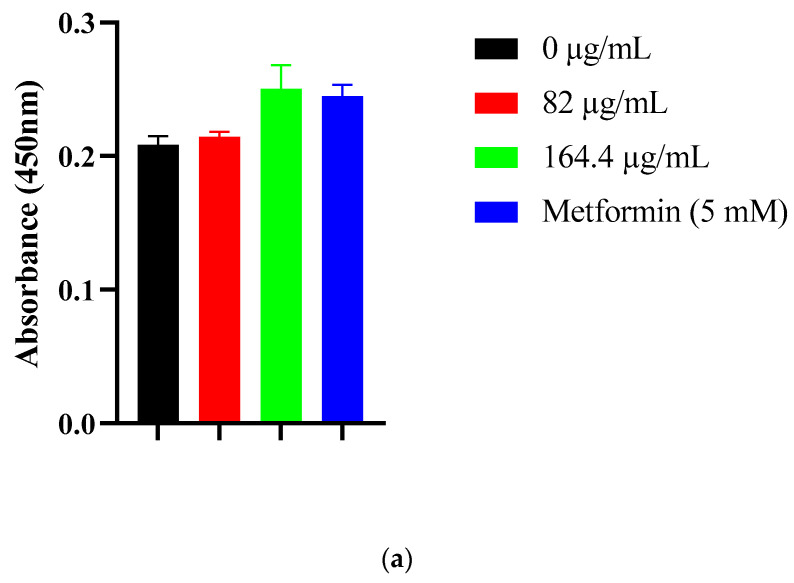

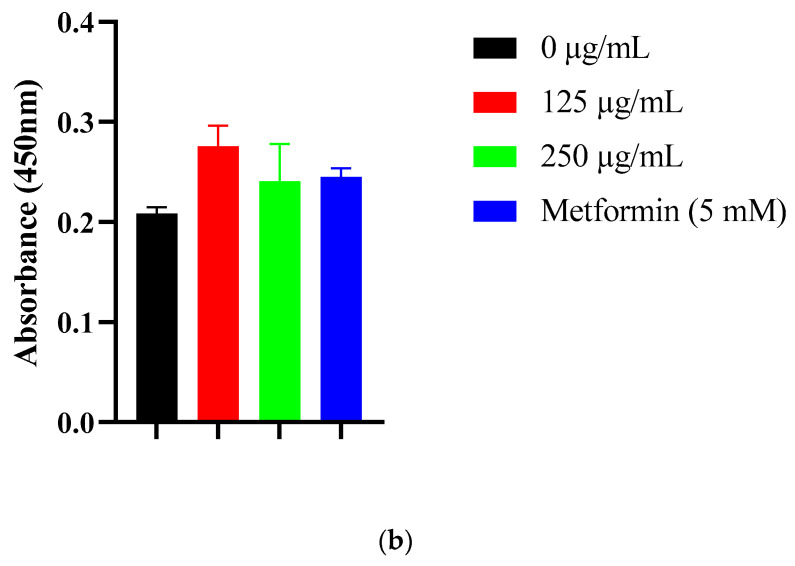
SGLT1 expression analysis in small intestinal epithelial cells following treatment with *S. birrea* leaf formulations. (**a**) Effects of CE treatment on SGLT1 expression over 60 min. (**b**) Effects of AgNP treatment on SGLT1 expression over 60 min. Data presented as mean ± SD (n = 3). No significant differences were observed (two-way ANOVA, *p* > 0.05).

As shown in Figure 3a, treatment with CE at concentrations of 82 and 164 µg/mL did not significantly alter SGLT1 expression levels at the 60 min time point. Expression remained at approximately 100% relative to baseline, comparable to untreated control cells.

Similarly, AgNP treatment at 125 and 250 µg/mL did not significantly affect SGLT1 expression levels (Figure 3b). Despite the transient elevation in extracellular glucose observed at 30 min with AgNP treatment, no corresponding changes in SGLT1 expression were detected.

### 2.8. DPP-IV Activity Inhibition

We evaluated the capacity of *S. birrea* formulations to inhibit DPP-IV, a clinically validated target for managing postprandial hyperglycemia through preservation of incretin hormones (GLP-1 and GIP) [13,14].

CE demonstrated no detectable DPP-IV inhibitory activity across all tested concentrations (82, 164.4, and 330 µg/mL), with inhibition remaining at 0 ± 0% (identical to untreated controls; Table 5). Consequently, an IC_50_ value could not be determined for CE, indicating that this preparation lacks significant DPP-IV inhibitory properties within the concentration range examined.

In striking contrast, AgNPs exhibited robust concentration-dependent inhibition (Table 5): 31.06 ± 0.34% at 125 µg/mL, 56.61 ± 0.15% at 250 µg/mL, and 72.45 ± 0.19% at 500 µg/mL (IC_50_: 220.5 µg/mL). While less potent than the positive control sitagliptin (90.41 ± 0.22% at 1 µM), AgNPs achieved substantial inhibition at concentrations below cytotoxic thresholds. To differentiate nanoparticle-mediated inhibition from potential ionic silver effects, AgNO_3_ (ionic silver) was tested at concentrations matched to the AgNP assay range (Table S4). AgNO_3_ produced negligible DPP-IV inhibition across all tested doses (0.89%, 0.98%, and 1.77% at 125, 250, and 500 μg/mL, respectively), indicating that dissolved/ionic silver alone cannot account for the robust inhibition observed with AgNPs. These data support that the marked DPP-IV inhibition is specific to the nanoformulation and is likely driven by nanoparticle surface/capping chemistry rather than Ag^+^ release.

### 2.9. Antioxidant Activities: Complementary Mechanisms

We evaluated antioxidant properties using multiple complementary assays to capture different mechanistic dimensions of radical scavenging and cellular protection.

#### 2.9.1. DPPH Radical Scavenging Activity

**Figure 4:**
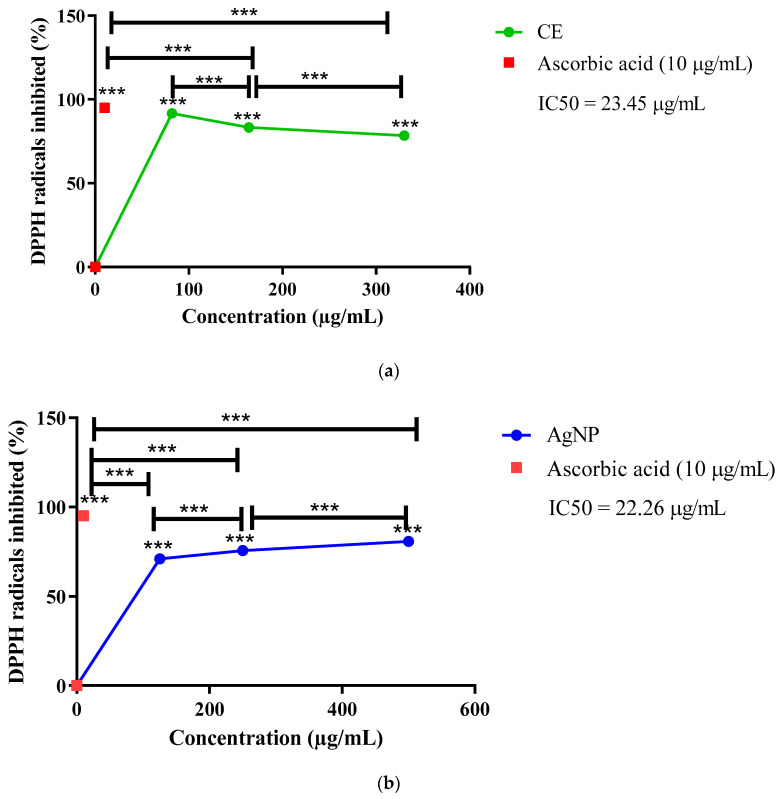
DPPH radical scavenging activity of *S. birrea* leaf preparations. (**a**) Dose–response curve for CE showing non-monotonic dose–response activity with maximal inhibition at lower concentrations. (**b**) Dose–response curve for AgNPs showing direct concentration-dependent activity. Data presented as mean ± SD (n = 3). IC_50_ values determined by nonlinear regression analysis. *** *p* < 0.001 vs. control; one-way ANOVA with Dunnett/Tukey.

Both CE and AgNPs demonstrated DPPH radical scavenging activity. Following calculation of IC_50_ values using nonlinear regression restricted to the monotonic response range (see Methods and Supplementary Materials), IC_50_ values were 23.45 µg/mL for CE and 22.26 µg/mL for AgNPs (Figure 4). These findings indicate broadly comparable radical scavenging potency between CE and AgNPs, with a slight numerical improvement following nanoformulation.

#### 2.9.2. Total Antioxidant Content (TAC)

**Figure 5:**
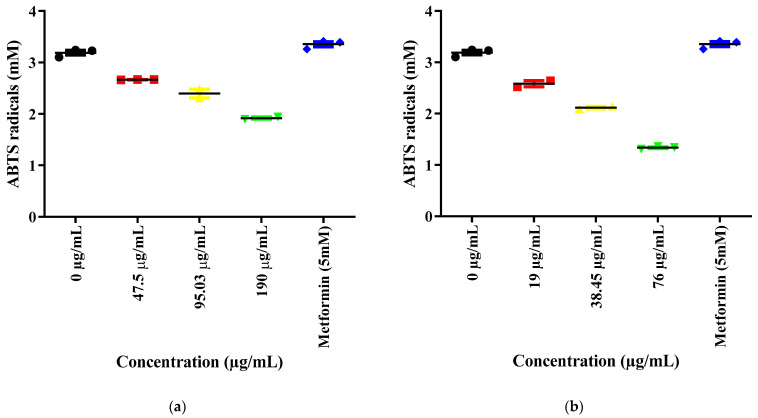
Total antioxidant capacity in kidney cells following treatment with *S. birrea* preparations. (**a**) TAC values (absorbance at 405 nm) for CE at various concentrations (n = 3) showing dose-dependent reduction in absorbance (increased antioxidant capacity); (**b**) TAC values for AgNPs at various concentrations (n = 3) showing greater dose-dependent reduction compared to CE. Note: Lower absorbance values indicate higher antioxidant capacity. Data presented as mean ± SD (n = 3). *p* < 0.001 vs. untreated control (one-way ANOVA with Tukey’s post hoc test).

In contrast to the DPPH results, AgNPs demonstrated superior total antioxidant content (TAC) activity when compared to CE, and this occurred in a dose-dependent manner (Figure 5). In this assay format, lower absorbance values indicate higher antioxidant content due to greater inhibition of ABTS^+^ formation [23,24].

The results indicate that both CE and AgNPs demonstrated dose-dependent antioxidant protection in kidney cell media. As the concentration of CE or AgNPs increased, the TAC values decreased (absorbance reduced), signifying an increase in antioxidant content. AgNPs were observed to be more potent in enhancing antioxidant content compared to CE at equivalent concentrations. Furthermore, both the untreated control (0 µg/mL) and metformin (5 mM) exhibited higher TAC absorbance values (indicating lower antioxidant content) relative to the treated groups.

#### 2.9.3. Cytoprotection Against Oxidative Stress in Kidney Cells

To assess functional antioxidant capacity under physiologically relevant conditions, we evaluated protection against H_2_O_2_-induced oxidative damage in HEK-293T kidney cells. This approach simulates conditions where antioxidant interventions must function in the presence of ongoing oxidative stress.

**Figure 6:**
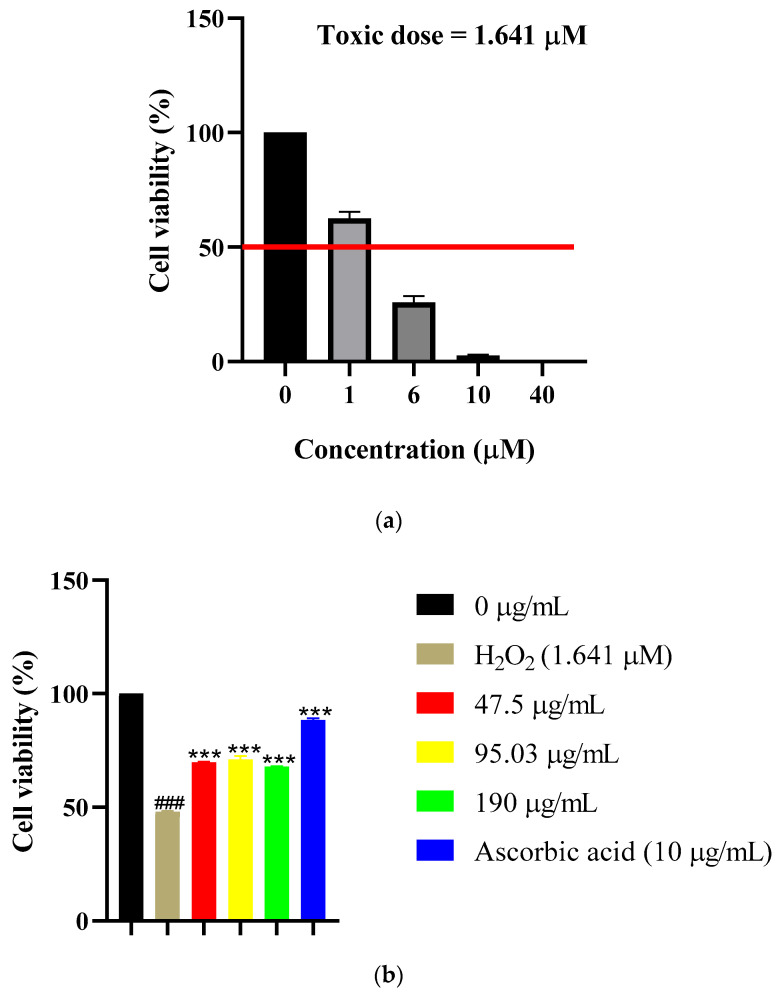

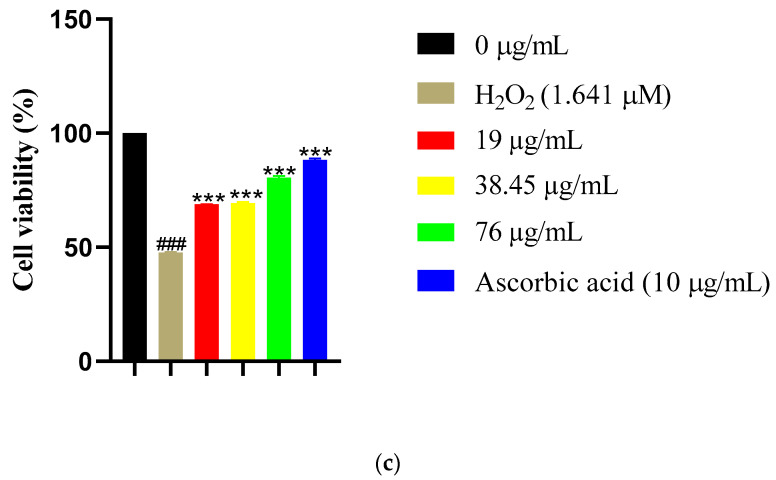
Cytoprotective effects against oxidative stress in HEK-293T kidney cells. (**a**) Dose–response curve for H_2_O_2_-induced cytotoxicity showing toxic dose = 1.641 μM. (**b**) Cytoprotective effects of CE against H_2_O_2_ (1.641 μM) at various concentrations. (**c**) Cytoprotective effects of AgNPs against H_2_O_2_ (1.641 μM) at various concentrations. Data presented as mean ± SD (n = 3). *p* < 0.001 vs. H_2_O_2_ alone (one-way ANOVA with Tukey’s post hoc test). ### *p* < 0.001 vs. untreated control; *** *p* < 0.001 vs. H_2_O_2_-only group (one-way ANOVA with Tukey’s post hoc test). The red horizontal line indicates the 50% viability threshold (IC50 criterion) used to guide the selection of cytotoxic working concentration.

Establishment of Oxidative Stress Model: HEK-293T cells were treated with serial dilutions of H_2_O_2_ (1–40 μM) for 3 h to establish dose–response relationships. A sigmoidal curve was observed, demonstrating concentration-dependent reduction in cell viability (Figure 6a). The toxic dose value was determined to be 1.641 μM H_2_O_2_, representing the concentration at which 50% cell viability was achieved. This established toxic dose value was subsequently employed in cytoprotection experiments to induce consistent and reproducible oxidative stress conditions.

CE provided modest cytoprotection (Figure 6b), maintaining viability at approximately 65–70% across all tested concentrations (47.5–190 µg/mL; *p* < 0.001 vs. H_2_O_2_ alone), compared to 50% viability with H_2_O_2_ treatment alone. Untreated control cells exhibited approximately 100% viability. Notably, none of the CE concentrations achieved complete restoration of cell viability to untreated control levels, suggesting partial but incomplete protection against oxidative stress during co-incubation conditions. The positive control, ascorbic acid (10 µg/mL), exhibited robust cytoprotective effects when co-incubated with H_2_O_2_, restoring cell viability to approximately 90% (*p* < 0.001 compared to H_2_O_2_ alone).

AgNPs demonstrated superior dose-dependent cytoprotection (Figure 6c): 65% viability at 19 µg/mL, 72.5% at 38.45 µg/mL, and approximately 80% at 76 µg/mL (*p* < 0.001 vs. H_2_O_2_ alone). Notably, AgNPs achieved greater protection at 2.5-fold lower concentrations than CE, suggesting enhanced bioavailability, more efficient ROS scavenging kinetics, or synergistic effects between the silver core and surface-bound bioactive compounds [19,20,25,26].

Comparison between CE and AgNP treatments under co-incubation conditions reveals that AgNPs exhibited superior protective effects at considerably lower concentrations. The CE at 190 µg/mL achieved approximately 67.5% viability, whereas AgNPs at 76 µg/mL achieved approximately 80% viability during simultaneous exposure to H_2_O_2_. Furthermore, AgNPs demonstrated a more pronounced dose–response relationship compared to CE, with progressive improvements in cell viability as concentration increased. In contrast, CE exhibited a plateau effect, with minimal differences observed between concentrations, suggesting potential saturation of protective mechanisms or insufficient potency to overcome the oxidative challenge at higher concentrations under co-incubation conditions.

## 3. Discussion

This study provides comprehensive evidence for the multifaceted anti-hyperglycemic and antioxidant properties of *S. birrea* leaf formulations, with biosynthesized AgNPs demonstrating enhanced and novel bioactivities compared to crude extract. Clinically, DPP-IV inhibitors such as sitagliptin improve incretin-mediated glycemic control and have been evaluated extensively in patients [57,58,59]. Green synthesis of spherical AgNPs (36.8 ± 8.6 nm) was achieved with well-defined physicochemical properties including face-centered cubic crystallinity (crystallite size: 32.1 nm), characteristic surface plasmon resonance (451 nm), and modest colloidal stability (zeta potential: −19.5 ± 8.36 mV) [39,40,55]. The discrepancy between hydrodynamic diameter (220 nm) and TEM-measured size reflects hydration layers, bioorganic capping agents, and particle association, typical features of phytochemical-stabilized nanoparticles [17,18]. FTIR analysis confirmed phytochemical coordination through shifted N-H vibrations, supporting a stabilization mechanism involving both steric and electrostatic components [15,16].

The moderate colloidal stability has implications for biological interpretation. Aggregation kinetics may reduce effective surface area, while protein corona formation in cell culture media alters nanoparticle behavior [17,18]. Nevertheless, reproducible bioactivity across assays suggests functional integrity within the experimental timeframe. Future optimization efforts should focus on enhancing zeta potential (target: ±30 mV) through pH adjustment, ionic strength modulation, or surface modification with additional capping agents while maintaining green synthesis principles [39].

The choice of cell line models represents an important consideration for interpreting the physiological relevance of the study findings. This study employed two established cell lines, Caco-2 cells (human colorectal adenocarcinoma) for intestinal modelling and HEK-293T cells (human embryonic kidney) for renal toxicity and cytoprotection assays. AgNPs demonstrated differential cytotoxicity across cell types: lower toxicity in intestinal cells (IC_10_: 250 µg/mL) but substantially higher toxicity in kidney cells (IC_10_: 38.45 µg/mL vs. 95.03 µg/mL for CE). This cell-type-specific cytotoxicity raises important safety concerns regarding systemic exposure and renal accumulation. The therapeutic window between efficacy and cytotoxicity appears narrow, particularly for kidney tissue, which is the target for oxidative protection in diabetic nephropathy. Several factors may contribute to differential cytotoxicity. Kidney cells may internalize AgNPs more efficiently than intestinal cells through specific endocytic pathways [19,20]. Intracellular AgNP dissolution releasing Ag^+^ ions may vary between cell types depending on pH, redox environment, and protein binding.

Beyond cytotoxicity studies, the current study provides valuable proof-of-concept data supporting the multi-target therapeutic potential of *S. birrea* formulations. The demonstrated enzyme inhibition, selective DPP-IV activity, and intracellular antioxidant delivery represent mechanistic insights that will guide more sophisticated validation studies in physiologically relevant model systems. Both *S. birrea* leaf CE and AgNPs potently inhibited α-amylase (CE IC_50_: 14 µg/mL; AgNPs IC_50_: 14.07 µg/mL) and α-glucosidase (CE IC_50_: 15.96 µg/mL; AgNPs IC_50_: 15.82 µg/mL), with activity comparable to acarbose when contextually interpreted [1,2,5]. The 8- to 14-fold higher IC_50_ values relative to acarbose should not diminish the therapeutic potential of these formulations, as plant extracts typically function at higher concentrations but may offer advantages including multi-component synergy, reduced tolerance development, and broader metabolic effects not captured by single IC_50_ comparisons [21,22].

Non-monotonic dose–response relationship with maximal inhibition at lower concentrations and progressive reductions at higher concentrations represents a finding requiring further mechanistic investigation. Potential explanations include optimal enzyme–inhibitor stoichiometry, concentration-dependent molecular aggregation, conformational enzyme changes, or assay interference [1,2,3]. Previous studies on *S. birrea* stem bark identified non-competitive α-glucosidase inhibition [49], consistent with allosteric binding that might explain saturation at higher concentrations. Kinetic analysis using Lineweaver–Burk plots would clarify inhibition modes and inform optimal therapeutic dosing. The observed non-monotonic pattern may reflect assay interference at higher concentrations; future work will confirm this using kinetics and turbidity/blank corrections.

Several plausible mechanisms may account for the observed modulation of enzyme activities mediated by the *S. birrea* CE and AgNPs. At higher concentrations (>200 µg/mL), polyphenolic compounds may directly interact with the chromogenic substrates (CNPG3 for α-amylase, pNPG for α-glucosidase) through non-covalent associations such as π-π stacking or hydrogen bonding. Both substrates contain aromatic chromophores that are prone to such interactions with polyphenolic compounds. This substrate sequestration would reduce substrate availability for enzyme-catalyzed hydrolysis, manifesting as apparent reduced inhibition in the spectrophotometric assay, which could be a measurement artifact rather than true loss of inhibitory activity.

At higher concentrations, flavonoids and tannins (major constituents identified in phytochemical screening, Table 2) are known to cause non-specific protein aggregation through multiple weak interactions [15,16]. This aggregation may sequester both enzyme and substrate into insoluble complexes or cause enzyme precipitation. Paradoxically, this could reduce measured inhibition by removing both enzyme and inhibitor from solution while simultaneously causing irreversible enzyme inactivation that would not be captured by our endpoint absorbance measurements. Excessive binding of polyphenols to non-active site regions (secondary binding sites) may induce conformational changes in the enzyme structure that partially restore catalytic activity. This phenomenon has been reported for tannin-rich plant extracts interacting with digestive enzymes, where high polyphenol concentrations can trigger protein conformational shifts that counteract active-site inhibition [34].

High extract concentrations increase solution turbidity and light scattering due to colloidal particles, aggregated polyphenols, and suspended material. This directly affects absorbance readings at 405 nm, potentially masking true enzyme activity. Additionally, highly colored extracts at elevated concentrations may contribute baseline absorbance that interferes with product detection. To definitively resolve the mechanism underlying this non-monotonic dose–response relationship with maximal inhibition at lower concentrations observed in the *S. birrea* CE and AgNPs, future experiments should include: (1) turbidity measurements at all test concentrations with appropriate corrections, (2) direct HPLC quantification of substrate availability in the presence of extracts, (3) comprehensive enzyme kinetics with Lineweaver–Burk analysis across the full concentration range to distinguish competitive, non-competitive, and mixed inhibition modes, (4) fluorescence-based enzyme assays to eliminate chromophore interference, and (5) dynamic light scattering to monitor aggregation kinetics during the assay.

Nanoparticle-mediated multivalent presentation of surface ligands can enhance target engagement and binding avidity compared with free phytochemicals [60,61,62,63]. The non-monotonic concentration–response behavior observed at higher concentrations in the α-amylase (CNPG3), α-glucosidase (pNPG), and DPPH assays is consistent with assay interference phenomena frequently encountered in natural product screening [64,65]. Colored/turbid extracts and phytochemical-capped nanoparticles can contribute background absorbance and light scattering at assay readout wavelengths, thereby biasing inhibition estimates even when sample blanks are applied [64,65].

In absorbance-based glycosidase assays (405 nm), incomplete correction can arise from wavelength overlap, time-dependent optical changes, and matrix effects that alter baseline absorbance across the reaction interval [64].

For DPPH, the canonical readout is ~517 nm; although absorbance was measured at 490 nm in this study (Methods), colored samples and scattering can still distort the apparent reduction signal, particularly at high sample loads [65].

Accordingly, IC_50_ values were calculated by nonlinear regression using only the monotonic portion of each curve (low-to-mid concentration range) where signal distortion is minimized. This approach is consistent with published protocols emphasizing robust blank correction for botanical extracts and with assay artifact mitigation guidance recommending exclusion of interference-dominated regions when estimating potency metrics [64,66,67].

Until these mechanistic studies are completed, we recommend calculating IC_50_ values using the ascending portion of dose–response curves (low-to-mid concentrations, typically <150 µg/mL) where specific enzyme–inhibitor interactions dominate over non-specific interference effects. The IC_50_ values reported in this study (Table 3 and Table 4) were calculated from this linear region and therefore represent the intrinsic inhibitory potency of the formulations. For therapeutic applications, optimal dosing would target these lower concentration ranges where inhibition is maximal and artifacts are minimal. Importantly, optimal inhibitory concentrations remained well below cytotoxic thresholds (IC_10_ = 164–250 µg/mL for intestinal cells), establishing a favorable therapeutic window for both formulations. This safety margin is particularly relevant for oral antidiabetic applications where intestinal cells represent the primary site of action.

Neither CE nor AgNPs significantly altered glucose uptake or SGLT1 expression in Caco-2 cells, indicating limited modulation of SGLT1 or GLUT2 transporters [6,7,8,9,10,11]. The transient glucose elevation observed with AgNPs at 30 min, followed by normalization, may reflect cellular stress responses, membrane perturbations, or metabolic adjustments [11,19,20]. These findings clarify that the anti-postprandial hyperglycemic potential operates primarily through reduced carbohydrate digestion (enzyme inhibition) rather than through direct intestinal glucose absorption inhibition. This mechanism complements rather than duplicates existing therapeutic approaches targeting insulin-sensitive tissues [4,5]. By reducing carbohydrate digestion in the intestinal lumen, these formulations decrease the glucose available for absorption, thereby attenuating postprandial glucose excursions without directly affecting insulin signaling pathways. This indirect approach may offer advantages in patients with impaired insulin sensitivity or β-cell dysfunction.

The most striking finding was selective DPP-IV inhibition by AgNPs (IC_50_: 220.5 µg/mL, 72.45% inhibition at 500 µg/mL), completely absent in CE (0% inhibition across all concentrations tested). This transformation represents a nanotechnology-emergent property, a pharmacological activity absent in the source material that appears exclusively upon nanoparticle formation and challenges the assumption that biosynthesized nanoparticles merely serve as delivery vehicles for phytochemical bioactivity. Although *Sclerocarya birrea* leaf AgNPs inhibited recombinant DPP-IV in a cell-free enzyme assay, inhibition in living human cells cannot be assumed because cellular environments introduce additional layers of complexity. In biological fluids and culture media, AgNPs rapidly acquire a protein corona that can alter surface presentation of phytochemical capping agents and reduce access to the catalytic site of membrane-associated or soluble DPP-IV. In addition, effective inhibition in cells would require that the bioactive moieties presented on the AgNP surface either (i) interact with extracellular DPP-IV at the plasma membrane or (ii) modulate DPP-IV activity indirectly through altered trafficking, shedding, or expression. Nevertheless, the emergence of DPP-IV inhibition after nanoformulation suggests that multivalent ligand presentation on the nanoparticle surface may enhance binding avidity compared with free phytochemicals, supporting the hypothesis that AgNPs could retain inhibitory activity in more complex systems. Future work should therefore validate DPP-IV inhibition in relevant cellular models. Such experiments will clarify whether the DPP-IV inhibition observed here translates to physiologically relevant conditions. DPP-IV is a homodimeric serine protease whose dimeric architecture and binding-site organization may permit geometry-dependent interactions that are less accessible to dispersed phytochemicals [62]. During green synthesis, polyphenolic compounds adsorb/coordinate onto the AgNP surface (supported by FTIR N–H shifts), potentially clustering ligands into nanoscale arrays that favor multivalent engagement. Such multivalency can increase apparent binding strength (avidity) by promoting rebinding and longer residence times, yielding substantial gains over monovalent interactions. [60,61,63]. In parallel, redox processes during Ag^+^ reduction may generate oxidized polyphenol derivatives (e.g., quinone-like species) with altered reactivity toward DPP-IV. Collectively, these non-exclusive hypotheses provide plausible explanations for the nanoparticle-emergent DPP-IV inhibition and motivate targeted validation (e.g., LC–MS/MS profiling and kinetic mode-of-inhibition studies).

The divergent antioxidant profiles across assays reveal complementary mechanisms optimized for different physiological contexts. DPPH radical scavenging potency was broadly comparable between formulations (CE IC_50_: 23.45 µg/mL; AgNPs IC_50_: 22.26 µg/mL), indicating that nanoformulation preserved antioxidant scavenging capacity, with CE showing higher maximal scavenging at the tested concentrations, reflecting abundant accessible hydroxyl groups for direct electron donation [23,24]. Conversely, AgNPs exhibited enhanced TAC and superior cytoprotection against H_2_O_2_-induced oxidative damage in kidney cells at 2.5-fold lower concentrations than CE [19,20,25,26]. This pattern of reduced extracellular radical scavenging but enhanced intracellular antioxidant activity has been consistently observed with green-synthesized AgNPs from diverse plant sources [53,54], suggesting an inherent trade-off during nanoformulation. Mechanistically, nanoparticle-mediated cellular uptake enables intracellular ROS modulation not captured by extracellular DPPH assays. Several mechanisms may contribute to enhanced intracellular activity: enhanced cellular uptake, sustained release and redox cycling. During enhanced cellular uptake, nanoparticles may be internalized via endocytosis. Therefore, these nanoparticles deliver antioxidant compounds directly to intracellular compartments where ROS are generated (e.g., mitochondria, endoplasmic reticulum) [19,20]. When phytochemicals bound to nanoparticle surfaces are released gradually within cells, prolonged antioxidant protection is provided via sustained release [25,26]. This may also result in redox cycling, whereby the silver core itself may participate in redox reactions, amplifying antioxidant capacity beyond that provided by phytochemicals alone. For therapeutic applications targeting intracellular oxidative stress in diabetic complications (particularly nephropathy), AgNPs may offer superior efficacy despite seemingly inferior DPPH performance [29,30,31]. The superior kidney cell protection by AgNPs is particularly relevant given that oxidative stress in renal tissue represents a key mechanism underlying diabetic complications. Chronic hyperglycemia-induced ROS production impairs podocyte function, promotes mesangial expansion, and drives glomerular basement membrane thickening, hallmarks of diabetic nephropathy [29,30,31].

Both formulations retained major phytochemical classes (flavonoids, tannins, alkaloids, terpenoids, glycosides, steroids, saponins), supporting multi-target bioactivity [32,33]. Carbohydrate absence in AgNPs suggests consumption as reducing agents during synthesis, consistent with the established mechanism of green nanoparticle synthesis where reducing sugars donate electrons to reduce Ag^+^ to Ag^0^ [40,55]. Additionally, removal of carbohydrates could reduce matrix interference and potentially unmask phytochemicals that interact with DPP-IV when presented multivalently on the AgNP surface. The detection of saponins in South African *S. birrea* contrasts with their reported absence in Malian samples [44], highlighting geographic variation driven by environmental factors (soil composition, pH, nutrient availability, water stress, temperature, altitude) that influence secondary metabolite biosynthesis [32,33]. This emphasizes the importance of specifying plant origin and validating bioactivity across geographic populations for standardization and quality control purposes. While qualitative screening provides useful compositional overview, it lacks the specificity and quantification necessary for mechanistic understanding [37,38]. Future studies employing HPLC-DAD-MS or LC-MS/MS would enable: (i) identification and quantification of specific bioactive compounds; (ii) determination of which compounds are lost or modified during synthesis; (iii) correlation of specific molecules with observed bioactivities, particularly the novel DPP-IV inhibition; and (iv) establishment of chemical markers for quality control and standardization.

## 4. Materials and Methods

### 4.1. Materials

All chemicals and reagents were of analytical grade. Dulbecco’s Modified Essential Medium (DMEM), penicillin/streptomycin (Pen/Strep), fetal calf serum (FCS), and trypsin were procured from Highveld Biological (Johannesburg, South Africa). Dimethyl sulfoxide (DMSO), bovine serum albumin (BSA), phosphate-buffered saline (PBS), silver nitrate (AgNO_3_), 2.2-diphenyl-1-picrylhydrazyl (DPPH), methanol (MeOH), and ethanol were obtained from Sigma-Aldrich (St. Louis, MO, USA) and Merck Chemicals (Johannesburg, South Africa). Metformin (Glucophage 500 mg) was obtained from Merck (Darmstadt, Germany). Acarbose, ascorbic acid, D-glucose and ferric chloride, were purchased from Sigma-Aldrich (St. Louis, MO, USA). DPP-IV enzyme (human recombinant), H-Gly-Pro-AMC substrate, and sitagliptin were used for enzyme inhibition assays. α-Amylase from porcine pancreas (Type VI-B), α-glucosidase from Saccharomyces cerevisiae (Type I), 2-chloro-4-nitrophenol α-D-maltotrioside (CNPG3), and p-nitrophenyl-α-D-glucopyranoside (pNPG) were obtained from Sigma-Aldrich (St. Louis, MO, USA).

### 4.2. Plant Material Collection and Identification

Fresh *S. birrea* leaves were collected in July 2023 from the Marula Pack House, located at 142 Main Road, Kwa Ngwanase 3973, KwaZulu-Natal, South Africa (GPS coordinates: 27°01′42.8″ S, 32°43′18.5″ E). The plant specimen was prepared for herbarium deposition, mounted by Mr. N. Khathi, and taxonomically identified by Dr. Syd Ramdhani from the School of Life Sciences, University of KwaZulu-Natal. The authenticated specimen was deposited in the Bews Herbarium (Pietermaritzburg Campus, University of KwaZulu-Natal) under the barcode number UDW23007 (Specimen ID: SH1).

### 4.3. Preparation of Leaf Extract

The collected *S. birrea* leaves were thoroughly washed with distilled water to remove surface contaminants and air-dried at room temperature (approximately 25 °C) for 14 days. Once dried, the leaves were ground into a fine powder using a hammermill (United Scientific, Gauteng, South Africa). Approximately 10 g of the powdered leaf material was boiled in 1000 mL of deionized water for 10 min with continuous stirring. The resulting mixture was filtered using Whatman No. 1 filter paper (GE Healthcare, Chicago, IL, USA) to remove insoluble residues.

The filtrate, representing the crude aqueous extract (CE) of *S. birrea* leaves, was divided into two portions: one portion was used directly for silver nanoparticle synthesis, and the remaining portion was freeze-dried using a VirTis BenchTop Pro freeze dryer (SP Scientific, Warminster, PA, USA) to obtain a powdered form. The freeze-dried CE was stored at −20 °C until further use. The extraction yield was calculated as follows:

Extraction yield (%) = (Weight of dried extract/Weight of dried plant material) × 100

Prior to experimental procedures, the powdered extract was reconstituted in PBS, filter-sterilized using 0.22 µm syringe filters (Millipore, Burlington, MA, USA), and diluted in fully supplemented cell culture media to achieve the desired working concentrations.

### 4.4. Synthesis and Characterization of Silver Nanoparticles

Silver nanoparticles (AgNPs) were synthesized using the aqueous *S. birrea* leaf CE as a reducing and capping agent, following established green synthesis protocols [40,55]. Briefly, 10 mL of the *S. birrea* leaf CE filtrate was added to 90 mL of 6 mM silver nitrate (AgNO_3_; Sigma-Aldrich) solution under continuous magnetic stirring at room temperature (25 °C). The mixture was incubated for 18 h in darkness to prevent photoreduction. A visible color change from yellowish-green to dark brown indicated the formation of *S. birrea* leaf AgNPs, consistent with surface plasmon resonance characteristic of silver nanoparticles [40,55].

The synthesized AgNPs were characterized using the following techniques:

UV–Visible Spectroscopy: Absorption spectra were recorded in the range of 300–700 nm using a UV-2600 spectrophotometer (Shimadzu, Kyoto, Japan) to confirm AgNP formation via surface plasmon resonance peak detection. The characteristic SPR peak was observed at 451 nm, confirming successful nanoparticle synthesis.

Fourier-Transform Infrared Spectroscopy (FTIR): FTIR spectra were obtained using a PerkinElmer Spectrum 100 FTIR spectrometer (PerkinElmer, Waltham, MA, USA) in the range of 4000–400 cm^−1^ to identify functional groups involved in AgNP stabilization [15,16]. Samples were prepared as KBr pellets (1% *w*/*w* sample in KBr) and scanned at a resolution of 4 cm^−1^ with 32 scans per spectrum.

X-Ray Diffraction (XRD): XRD analysis was performed using a Bruker D8 Advance diffractometer (Bruker, Billerica, MA, USA) with Cu Kα radiation (λ = 1.5406 Å) operating at 40 kV and 40 mA. Diffraction patterns were recorded over a 2θ range of 20° to 80° with a step size of 0.02° and a counting time of 1 s per step. Peak positions were indexed by comparison to the JCPDS card no. 04-0783 for face-centered cubic (FCC) silver.

Crystallite size was calculated using the Scherrer equation applied to the most intense (111) peak:

D = Kλ/(β cos θ)

where D is the crystallite size, K is the shape factor (0.9 for spherical particles), λ is the X-ray wavelength (1.5406 Å), β is the full width at half maximum (FWHM) in radians after correction for instrumental broadening, and θ is the Bragg angle. FWHM values were determined using OriginPro 2024 software with Gaussian peak fitting. Instrumental broadening was determined using a LaB_6_ standard (NIST SRM 660a; Lanthanum Hexaboride Powder, Line Position and Line Shape Standard for Powder Diffraction; National Institute of Standards and Technology, Gaithersburg, MD, USA; certificate issue date 13 September 2000) and corrected using the formula:

β^2^sample = β^2^measured − β^2^instrument

The calculated crystallite size (32.1 nm from the (111) peak) differs from the TEM-determined average particle size (36.8 nm) due to: (1) XRD measuring individual crystal domain size within particles, (2) TEM measuring overall particle size including surface-bound organic material, and (3) potential polycrystallinity where individual particles may comprise multiple crystal domains.

Transmission Electron Microscopy (TEM): Particle morphology and core size distribution were determined using transmission electron microscopy. TEM images were acquired using a JOEL 1010 transmission electron microscope (JEOL Ltd., Tokyo, Japan). Samples were prepared by depositing a drop of diluted AgNP suspension onto carbon-coated copper grids, allowing the grids to air-dry at room temperature prior to imaging. Particle size distribution was determined by measuring ≥100 nanoparticles using ImageJ Particle size distribution was determined by measuring ≥100 nanoparticles using ImageJ v1.53k (National Institutes of Health, Bethesda, MD, USA).

Scanning Electron Microscopy (SEM): Surface morphology was examined using a Zeiss Sigma 300 VP field emission scanning electron microscope (Carl Zeiss, Oberkochen, Germany) at an accelerating voltage of 5 kV and working distance of 8–10 mm. Samples were prepared by depositing a drop of AgNP suspension onto silicon wafers, allowing to dry, and then sputter-coating with gold (10 nm thickness) using a Quorum Q150R ES sputter coater to enhance conductivity and image quality.

Dynamic Light Scattering (DLS) and Zeta Potential: Hydrodynamic diameter and surface charge were measured using a Malvern Zetasizer Nano ZS (Malvern Panalytical, Malvern, UK) at 25 °C [39]. For DLS measurements, AgNP samples were diluted 1:100 in deionized water (filtered through 0.22 µm filters), and measurements were performed in disposable polystyrene cuvettes with three measurements per sample. For zeta potential measurements, samples were diluted in 10 mM KCl solution and analyzed in folded capillary cells (DTS1070) with automatic voltage optimization.

Following synthesis and characterization, the reaction mixture was centrifuged at 15,000× *g* for 20 min at 4 °C using a Hermle Z326K refrigerated centrifuge (Hermle Labortechnik, Wehingen, Germany) to pellet the AgNPs. The supernatant was discarded, and the pellet was resuspended in deionized water. This washing procedure was repeated three times to remove unreacted AgNO_3_ and unbound biomolecules. The purified AgNPs were then freeze-dried and stored at −20 °C in light-protected containers. Prior to experimental applications, the *S. birrea* leaf AgNPs were dispersed in 1% DMSO, diluted with PBS, and filter-sterilized (0.22 µm). Working concentrations were prepared by diluting the nanoparticle suspension in fully supplemented cell culture media, ensuring that final DMSO concentration did not exceed 0.1% (*v*/*v*).

### 4.5. Cell Culture

Caco-2 cells (human colorectal adenocarcinoma, ATCC HTB-37) and HEK-293T cells (human embryonic kidney, ATCC CRL-3216) were obtained from Cellonex (Johannesburg, South Africa). Cells were cultured in Dulbecco’s Modified Eagle Medium (DMEM; Highveld Biological, Johannesburg, South Africa) supplemented with 10% (*v*/*v*) heat-inactivated fetal calf serum (FCS), 2 mM L-glutamine, 100 U/mL penicillin, and 100 µg/mL streptomycin. Cells were maintained at 37 °C in a humidified atmosphere containing 5% CO_2_ (Heracell 150i CO_2_ incubator, Thermo Scientific, Waltham, MA, USA) and subcultured upon reaching 80–90% confluency using 0.25% trypsin-EDTA. Cells between passages 15 and 25 were used for all experiments to ensure consistency and reproducibility [6,7,8].

### 4.6. Cytotoxicity Assay

To establish safe working concentrations and assess potential cytotoxic effects, cell viability was evaluated using the CellTiter-Glo^®^ Luminescent Cell Viability Assay (Promega, Madison, WI, USA), which quantifies intracellular ATP as a marker of metabolically active cells.

Caco-2 and HEK-293T cells were seeded into white-walled 96-well plates (Corning, NY, USA) at a density of 1 × 10^4^ cells/well in 100 µL complete DMEM and incubated overnight (16–18 h) to allow cell attachment. The following day, medium was aspirated and replaced with fresh medium containing serial dilutions of either *S. birrea* leaf CE (50–3000 µg/mL) or AgNPs (50–3000 µg/mL). Treatment stocks were prepared by dissolving freeze-dried CE in PBS or resuspending AgNPs in 1% DMSO with PBS, followed by filter sterilization (0.22 µm). Final DMSO concentration in culture medium did not exceed 0.1% (*v*/*v*) to avoid solvent-mediated cytotoxicity. Untreated cells receiving medium alone served as negative controls (100% viability reference).

After 24 h of exposure, 100 µL of CellTiter-Glo^®^ reagent was added directly to each well containing 100 µL culture medium (1:1 ratio). Plates were placed on an orbital shaker at room temperature for 2 min to induce cell lysis and stabilize the luminescent signal. Luminescence was measured using a GloMax^®^ Explorer Multimode Microplate Reader (Promega, Madison, WI, USA) with an integration time of 1 s per well.

Data Analysis: Background luminescence (medium without cells) was subtracted from all readings. Cell viability was calculated as:

% Viability = (Luminescence_treated_/Luminescence_control_) × 100

Dose–response curves were generated by plotting % viability against log_10_[concentration], and IC_10_ values (concentration causing 10% viability reduction) were determined using nonlinear regression (log[inhibitor] vs. normalized response, variable slope, four parameters) in GraphPad Prism 8.0 (GraphPad Software, La Jolla, CA, USA). The IC_10_ threshold was selected rather than IC_50_ to establish safe working concentrations for functional assays, ensuring ≥90% cell viability to minimize confounding effects of cytotoxicity on enzyme activity or glucose transport measurements. All experiments were performed in technical triplicates and repeated in three independent biological replicates (n = 3). Data are presented as mean ± standard deviation (SD). Subsequent functional assays utilized concentrations at or below 75% of the determined IC_10_ values.

### 4.7. α-Amylase Inhibition Assay

The α-amylase inhibitory activity of *S. birrea* leaf CE and AgNPs was assessed using 2-chloro-4-nitrophenol α-D-maltotrioside (CNPG3) as a chromogenic substrate [4,56]. Porcine pancreatic α-amylase catalyzes the hydrolysis of CNPG3, releasing 2-chloro-4-nitrophenol (CNP), which absorbs strongly at 405 nm. Inhibitors reduce CNP production, resulting in decreased absorbance proportional to inhibitory potency [1,4].

Assays were conducted in clear flat-bottom 96-well microplates (Corning, NY, USA). Each reaction mixture (total volume 150 µL) contained: 50 µL porcine pancreatic α-amylase (1 U/mL in 20 mM sodium phosphate buffer, pH 6.9, containing 6 mM NaCl; Sigma-Aldrich), 50 µL test sample (*S. birrea* leaf CE at 82, 164.4, and 330 µg/mL; AgNPs at 125, 250, and 500 µg/mL), and 50 µL CNPG3 substrate (2 mM final concentration in sodium phosphate buffer; Sigma-Aldrich). The reaction was initiated by substrate addition, and plates were incubated at 37 °C for 30 min in a temperature-controlled incubator. Absorbance at 405 nm was measured using a GloMax^®^ Explorer Multimode Microplate Reader.

Controls: Negative control (100% enzyme activity): α-amylase + PBS (replacing test sample) + substrate; Blank (background absorbance): PBS + test sample + substrate (no enzyme); Positive control: Acarbose (2.5 µg/mL; Sigma-Aldrich), a clinically used α-amylase/α-glucosidase inhibitor.

Data Analysis: Percentage inhibition of α-amylase activity was calculated as:

% Inhibition = [(A_405 control_ − A_405 sample_)/A_405 control_)] × 100

where A_405 control_ represents the absorbance of the negative control (no inhibitor), and A_405 sample_ represents the absorbance of the test sample. IC_50_ values (concentration producing 50% inhibition) were determined using nonlinear regression analysis (log[inhibitor] vs. response, variable slope, four parameters) in GraphPad Prism 8.0. All reactions were performed in technical triplicates across three independent experiments (n = 3). Data are presented as mean ± SD. Statistical comparisons between groups were performed using one-way analysis of variance (ANOVA) followed by Tukey’s post hoc test, with *p* < 0.05 considered statistically significant.

### 4.8. α-Glucosidase Inhibition Assay

The α-glucosidase inhibitory activity was evaluated using p-nitrophenyl-α-D-glucopyranoside (pNPG) as a chromogenic substrate [5,34,56]. α-Glucosidase from Saccharomyces cerevisiae hydrolyzes pNPG, releasing p-nitrophenol, a yellow product with maximum absorbance at 405 nm. Inhibitors reduce p-nitrophenol formation proportionally to their potency [5,34].

Assays were performed in 96-well microplates with the following reaction composition (total volume 150 µL): 50 µL α-glucosidase (1 U/mL in 100 mM potassium phosphate buffer, pH 6.8; Sigma-Aldrich), 50 µL test sample (*S. birrea* leaf CE at 82, 164.4, and 330 µg/mL; AgNPs at 125, 250, and 500 µg/mL), and 50 µL pNPG substrate (2 mM final concentration in potassium phosphate buffer; Sigma-Aldrich). Reactions were initiated by substrate addition, incubated at 37 °C for 30 min, and terminated by adding 50 µL of 1 M sodium carbonate (Na_2_CO_3_). Absorbance at 405 nm was immediately measured using a GloMax^®^ Explorer Multimode Microplate Reader.

Controls: Negative control: α-glucosidase + PBS + substrate (100% enzyme activity); Blank: PBS + test sample + substrate (no enzyme); Positive control: Acarbose (2.5 µg/mL).

Data Analysis: Percentage inhibition and IC_50_ values were calculated as described for α-amylase (Section 4.7). All experiments were performed in technical triplicates with three independent repeats (n = 3). Statistical analysis used one-way ANOVA with Tukey’s post hoc test (*p* < 0.05).

### 4.9. Extracellular Glucose Concentration Assay in Caco-2 Cells

To assess whether *S. birrea* preparations modulate intestinal glucose dynamics, glucose concentrations in Caco-2 cell culture medium were measured over time using the glucose oxidase-peroxidase (GOD-POD) colorimetric assay [6,7,8].

Caco-2 cells were seeded into 96-well plates at a density of 1 × 10^4^ cells/well in 100 µL complete DMEM and incubated overnight (16–18 h) at 37 °C with 5% CO_2_. Medium was then replaced with fresh DMEM containing 5.5 mM D-glucose (representing physiological postprandial concentration) supplemented with: *S. birrea* leaf CE (82, 164 µg/mL), *S. birrea* leaf AgNPs (125, 250 µg/mL), metformin (5 mM; positive control; Merck), or PBS (untreated control). Concentrations were selected based on cytotoxicity data (≤75% of IC_10_ values).

Glucose Measurement: At 0, 30, and 60 min, 10 µL of culture medium was collected and immediately assayed for glucose using the GOD-POD assay kit (GAGO20; Sigma-Aldrich) according to the manufacturer’s instructions. Briefly, 10 µL sample was mixed with 200 µL GOD-POD reagent in a fresh 96-well plate and incubated at 37 °C for 30 min, and absorbance was measured at 540 nm using a GloMax^®^ Explorer Multimode Microplate Reader. Glucose concentration was calculated from a standard curve prepared using D-glucose standards (0–10 mM) analyzed in parallel. Values are expressed as percentage relative to time 0 baseline for each treatment group to normalize for any inter-well variations in initial glucose concentration.

Data Analysis: Data are presented as mean ± SD (n = 3 independent experiments, each with technical triplicates). Two-way ANOVA with Bonferroni post hoc test was used to compare glucose concentrations across treatment groups and time points, with *p* < 0.05 considered statistically significant.

### 4.10. Quantification of SGLT1 in Caco-2 Cells

Cell culture, treatments, and controls. Caco-2 cells were cultured as described in Section 4.5 and seeded to reach 80–90% confluence before treatment. Cells were treated for 60 min at 37 °C and 5% CO_2_ with *S. birrea* leaf crude extract (CE; 82, 164 µg/mL), AgNPs (125, 250 µg/mL), metformin (5 mM, context control), or media only (untreated control). Where applicable, a DMSO vehicle control was included; final DMSO ≤ 0.1% (*v*/*v*) in all wells. All treatments were performed in biological triplicates (n = 3).

Sample collection and lysis. Immediately after treatment, plates were placed on ice and washed twice with ice-cold PBS. Cells were lysed on ice with RIPA buffer (Merck/Sigma-Aldrich R0278; 50 mM Tris-HCl pH 8.0, 150 mM NaCl, 1% NP-40/Igepal CA-630, 0.5% sodium deoxycholate, 0.1% SDS) supplemented with protease inhibitor cocktail (Merck P8340) and phosphatase inhibitor cocktails (Merck P2850, P5726). Lysates were scraped, incubated on ice 10–20 min, and clarified (12.000× *g*, 10 min, 4 °C); supernatants were collected for ELISA. Where storage was necessary, aliquots were kept at −80 °C and thawed once for analysis.

SGLT1 was quantified using the Human SGLT1 ELISA (Cloud-Clone Corp., SEE381Hu, manual E94381Hu). This is a double-antibody sandwich format using a pre-coated anti-SGLT1 microplate, biotin-conjugated anti-SGLT1 (Detection Reagent A), avidin–HRP (Detection Reagent B), TMB substrate, and acid stop for measurement at 450 ± 10 nm.

Detection range: 0.156–10 ng/mL; LOD: <0.057–0.060 ng/mL; precision: intra-assay CV <10%, inter-assay CV <12%. Standards and samples were run in duplicate and quantified by 4PL fits at 450 nm (±10 nm).

### 4.11. Dipeptidyl Peptidase-IV (DPP-IV) Inhibitory Assay

The DPP-IV inhibitory activity of *S. birrea* leaf CE and AgNPs was assessed using H-Gly-Pro-AMC as the substrate [13,14].

Assays were conducted in 96-well microplates. Each reaction mixture (total volume 150 µL) contained 50 µL of DPP-IV enzyme (human recombinant, 0.05 U/mL in 50 mM Tris-HCl buffer, pH 8.0; Sigma-Aldrich) and 50 µL of test sample (*S. birrea* leaf CE at 82, 164.4, and 330 µg/mL; AgNPs at 125, 250, and 500 µg/mL), preincubated at 37 °C for 10 min to allow enzyme–inhibitor interaction. The reaction was initiated by adding 50 µL of 2 mM H-Gly-Pro-AMC substrate (Sigma-Aldrich). After 30 min incubation at 37 °C, the reaction was stopped by adding 50 µL of 1 M sodium acetate buffer (pH 4.0). The release of AMC was quantified by measuring fluorescence (FLU, λ_ex_ = 360/λ_em_ = 460 nm) using a GloMax^®^ Explorer Multimode Microplate Reader.

Controls: Negative control (100% enzyme activity): DPP-IV + PBS + substrate; Blank: PBS + test sample + substrate (no enzyme); Positive control: Sitagliptin (1 µM; Sigma-Aldrich), a clinically approved DPP-IV inhibitor.

Data Analysis: Percentage inhibition of DPP-IV activity was calculated as:

% Inhibition = [(FLU _sample λex=360/λem=460_ − FLU _blank λex=360/λem=460_)/[(FLU _control λex=360/λem=460_ − FLU_blank λex=360/λem=460_] × 100

IC_50_ values were determined using nonlinear regression analysis in GraphPad Prism 8.0. All reactions were performed in technical triplicates across three independent experiments (n = 3). Data are presented as mean ± SD. Statistical comparisons used one-way ANOVA with Tukey’s post hoc test (*p* < 0.05).

### 4.12. Antioxidant Assays

#### 4.12.1. DPPH Radical Scavenging Assay

The DPPH radical scavenging activity of *S. birrea* leaf CE and AgNPs was determined using a modified method [23,24]. The assay measures the capacity of antioxidants to donate electrons or hydrogen atoms to the stable DPPH• radical (2.2-diphenyl-1-picrylhydrazyl), resulting in a color change from purple to yellow.

In a 96-well plate, 50 µL of test sample (*S. birrea* leaf CE or AgNPs at 82, 164.4, and 330 µg/mL for CE; 125, 250, and 500 µg/mL for AgNPs) was added to 150 µL of 0.1 mM DPPH solution (in methanol; Sigma-Aldrich). The mixture was incubated in the dark for 30 min at room temperature (25 °C) to prevent light-induced degradation of DPPH. Absorbance was measured at 490 nm using a GloMax^®^ Explorer Multimode Microplate Reader. Absorbance was read at 490 nm. To ensure comparability with the canonical 517 nm readout, we (i) performed blank subtraction (sample + methanol, no DPPH) at each concentration to correct for extract/nanoparticle color and turbidity and (ii) validated linearity and equivalence versus 517 nm using ascorbic acid controls in preliminary runs.

Controls: Blank: Methanol + test sample (no DPPH); Negative control: DPPH + PBS (no antioxidant); Positive control: Ascorbic acid (10 µg/mL; Sigma-Aldrich).

Data Analysis: The percentage scavenging activity was calculated as:

% Scavenging = [(A_490 control_ − A_490 sample_)/A_490 control_] × 100

IC_50_ values were determined using nonlinear regression in GraphPad Prism 8.0. All experiments were performed in technical triplicates across three independent experiments (n = 3). Data are presented as mean ± SD.

#### 4.12.2. Total Antioxidant Content (TAC) Assay

The total antioxidant content of *S. birrea* leaf CE and AgNPs was determined using the Sigma-Aldrich TAC assay kit, which is based on the oxidation of ABTS (2.2′-azino-bis(3-ethylbenzthiazoline-6-sulfonic acid)) by a ferryl myoglobin radical. This radical is generated from metmyoglobin in the presence of hydrogen peroxide. Antioxidant compounds inhibit the formation of the green-colored ABTS^+^ radical cation, and the resulting decrease in absorbance at 405 nm is proportional to the antioxidant content of the sample [23,24].

In a 96-well plate, 10 µL of test sample (*S. birrea* leaf CE or AgNPs at 47.5, 95.03, and 190 µg/mL for CE; 19, 38.45, and 76 µg/mL for AgNPs) was combined with 190 µL of the reagent solution mixture provided in the kit. The mixture was incubated at room temperature for the duration specified by the manufacturer. After incubation, absorbance was measured at 405 nm using a GloMax^®^ Explorer Multimode Microplate Reader. Lower absorbance values indicate higher antioxidant content due to greater inhibition of ABTS^+^ formation.

Note on Interpretation: In this assay, lower absorbance values indicate higher antioxidant capacity due to greater reduction of ABTS/myoglobin H_2_O_2_, which depletes the colored complex formed in the assay. Total antioxidant capacity was expressed as ascorbic acid equivalents (µg/mL) based on a standard curve. All experiments were performed in technical triplicates across three independent experiments (n = 3). Data are presented as mean ± SD.

#### 4.12.3. Hydrogen Peroxide (H_2_O_2_) Cytoprotection Assay

The cytoprotective effects of *S. birrea* leaf CE and AgNPs against oxidative stress-induced cellular injury were evaluated in HEK-293T cells using hydrogen peroxide (H_2_O_2_) as an oxidative stressor [29,31].

HEK-293T cells were seeded into 96-well plates at 1 × 10^4^ cells/well in 100 µL complete DMEM and incubated overnight. Cells were then treated with various doses of H_2_O_2_ (1–40 μM; Sigma-Aldrich) for 3 h to establish dose–response relationships. Cell viability was assessed using the CellTiter-Glo^®^ assay as described in Section 4.6. The toxic dose value for H_2_O_2_ was determined to be 1.641 μM (50% viability) using nonlinear regression analysis and was subsequently used in cytoprotection experiments.

HEK-293T cells were co-incubated with H_2_O_2_ (1.641 μM) and varying concentrations of either *S. birrea* leaf CE (47.5, 95.03, and 190 µg/mL) or AgNPs (19, 38.45, and 76 µg/mL) for 24 h. Ascorbic acid (10 µg/mL) served as a positive control. Cell viability was assessed using the CellTiter-Glo^®^ assay as described in Section 4.6.

Cytoprotective effects were calculated as the percentage of cell viability compared to untreated control (100% viability). Data are presented as mean ± SD (n = 3 independent experiments, each with technical triplicates). Statistical comparisons were performed using one-way ANOVA with Tukey’s post hoc test (*p* < 0.05).

### 4.13. Qualitative Phytochemical Screening

Qualitative phytochemical screening of *S. birrea* leaf CE and AgNPs was performed to identify the presence or absence of various secondary metabolites, including flavonoids, saponins, tannins, glycosides, terpenoids, carbohydrates, steroids, and alkaloids, using established colorimetric and precipitation methods [32,33,37].

Flavonoids Test: To 500 µL of either *S. birrea* leaf CE or AgNPs, 500 µL of 10% sodium hydroxide (NaOH) solution was added. The appearance of a yellow coloration, which turned colorless upon addition of 500 µL of 1 M hydrochloric acid (HCl), indicated the presence of flavonoids.

Saponins Test: 500 µL of either *S. birrea* leaf CE or AgNPs was vigorously shaken with 5 mL of distilled water in a 15 mL tube. The mixture was vortexed for 30 s and allowed to stand undisturbed for 10 min. The formation of a stable froth or foam (>1 cm height persisting for >15 min) was considered a positive indication of saponins.

Tannins and Phenols Test: To 500 µL of either *S. birrea* leaf CE or AgNPs, 100 µL of 2% ferric chloride (FeCl_3_) solution was added. The appearance of a blue-black or green-black precipitate confirmed the presence of tannins and phenolic compounds.

Carbohydrates Test: 500 µL of either *S. birrea* leaf CE or AgNPs was treated with 200 µL of Benedict’s reagent and heated in a boiling water bath for 5 min. The formation of a reddish-brown precipitate indicated the presence of reducing sugars.

Glycosides Test: To 500 µL of either *S. birrea* leaf CE or AgNPs, 200 µL of chloroform and 200 µL of glacial acetic acid were added. The mixture was cooled on ice for 5 min, followed by the addition of 150 µL of concentrated H_2_SO_4_. A green coloration was indicative of the presence of a steroidal nucleus, suggesting the presence of glycosides.

Steroids Test: 500 µL of either *S. birrea* leaf CE or AgNPs was mixed with 200 µL of chloroform and 150 µL of concentrated H_2_SO_4_. The development of a red coloration in the lower chloroform layer was taken as evidence of steroidal compounds.

Alkaloids Test: 500 µL of either *S. birrea* leaf CE or AgNPs was acidified with 500 µL of 1% hydrochloric acid (HCl) and heated in a boiling water bath for 5 min. After cooling, four drops of Dragendorff’s reagent (Sigma-Aldrich) were added. The appearance of turbidity or an orange-red precipitate indicated the presence of alkaloids.

Terpenoids Test: To 500 µL of either *S. birrea* leaf CE or AgNPs, 2 mL of chloroform was added and vigorously shaken. The chloroform layer was then separated, and 3 mL of concentrated H_2_SO_4_ was carefully added along the sides of the test tube. The formation of a reddish-brown coloration at the interface indicated the presence of terpenoids.

### 4.14. Statistical Analysis

All experiments were performed in technical triplicates and repeated independently at least three times (n = 3 biological replicates unless otherwise stated). Data are presented as mean ± standard deviation (SD). Statistical analysis was performed using GraphPad Prism software (Version 8.0, GraphPad Software, La Jolla, CA, USA). Normality of data distribution was assessed using the Shapiro–Wilk test. For comparisons between multiple groups, one-way analysis of variance (ANOVA) followed by Tukey’s post hoc test was used for normally distributed data. For time-course experiments (glucose dynamics assay), two-way ANOVA with Bonferroni post hoc test was employed to assess effects of treatment and time. A *p*-value < 0.05 was considered statistically significant. Dose–response curves and IC_50_/IC_10_ values were determined using nonlinear regression analysis (log[inhibitor] vs. normalized response, variable slope, four parameters model) in GraphPad Prism 8.0. IC_50_ values for α-amylase, α-glucosidase, and DPPH were calculated by nonlinear regression using only the monotonic portion of each concentration–response curve; non-monotonic regions at extreme concentrations (consistent with matrix/optical interference; see Supplementary Materials) were excluded from curve fitting.

## 5. Conclusions

This in vitro study provides comprehensive evidence for the multifaceted direct and indirect antihyperglycemic and antioxidant potential of *Sclerocarya birrea* leaf crude extract (CE) and biosynthesized silver nanoparticles (AgNPs).

Our findings align with and complement prior work on *S. birrea* leaves. Notably, Maharaj et al. (2022) identified antidiabetic flavonoids from an aqueous *S. birrea* leaf extract—myricetin, myricetin-3-O-β-D-glucuronide, and quercetin-3-O-β-D-glucuronide—and showed that these compounds stimulate 2-deoxyglucose uptake in differentiated C2C12 myocytes, with myricetin-3-O-β-D-glucuronide highlighted as a key bioactive constituent [68]. In contrast, the present study expands the mechanistic basis for the antidiabetic potential of *S. birrea* leaf formulations by demonstrating multi-target postprandial mechanisms (α-amylase/α-glucosidase inhibition), a nanoformulation-emergent DPP-IV inhibitory activity, and cellular cytoprotection against oxidative stress, providing additional translational rationale for developing leaf-derived products and nano-enabled phytomedicines for T2DM management.

## 6. Limitations

All experiments in this study were conducted in vitro, which may limit the extrapolation of findings to vivo systems.

In addition, SEM–EDX elemental analysis was not performed in this study; therefore, direct elemental confirmation (e.g., Ag wt%/at%) is not provided and will be included in follow-up work. Furthermore, Ag^+^ dissolution (release of dissolved silver species) from AgNPs was not quantified in the assay buffers; therefore, we cannot fully apportion observed bioactivity to nanoparticle-associated versus dissolved ionic silver contributions under the tested conditions.

Additionally, the molecular mechanisms underlying the observed non-monotonic dose–response relationship will be investigated in the future.

## Figures and Tables

**Table 1:** Summary of AgNP Physicochemical Characterization Parameters.

Parameter	Value/Description
UV–Vis SPR Peak	451 nm
TEM Average Size	36.8 ± 8.6 nm (range: 22–50 nm)
Morphology	Predominantly spherical with agglomeration
XRD Crystallite Size	32.1 nm (Debye–Scherrer)
Crystal Structure	FCC, 2θ: 37.93°, 44.08°, 64.10°, 76.97°, 81.07°
DLS Hydrodynamic Diameter	220 nm
PDI	0.334 (moderate polydispersity)
Zeta Potential	−19.5 ± 8.36 mV
FTIR Key Peaks	O-H (3653 cm^−1^), N-H (3224 cm^−1^), C-H (2917 cm^−1^), C=O (1604 cm^−1^)

**Table 2:** Qualitative Phytochemical Screening of *S. birrea* Leaf Formulations.

	*S. birrea* Leaf CE	*S. birrea* Leaf AgNP
Flavonoids	+	+
Saponins	+	+
Tannins	+	+
Glycosides	+	+
Terpenoids	+	+
Carbohydrates	+	−
Steroids	+	+
Alkaloids	+	+

+ Presence; − Absence.

**Table 3:** α-Amylase Inhibitory Activity of *S. birrea* Formulations. Data are presented as mean ± SD from three independent experiments (n = 3), each performed with three technical replicates (total 9 measurements per condition). Different superscript letters (a, b, c, d, e) indicate statistically significant differences between groups (*p* < 0.05, one-way ANOVA with Tukey’s post hoc test for multiple comparisons). IC_50_ values were calculated by nonlinear regression analysis (log[concentration] vs. response, variable slope four-parameter model) using GraphPad Prism 8.0 software. CE, crude extract; AgNPs, silver nanoparticles.

Treatment Group	Concentration (µg/mL)	α-Amylase Inhibited (%)	IC_50_ (µg/mL)
Untreated	0	0 ± 0 ^a^	
CE	82	87.22 ± 0.27 ^b^	14
	164.4	81.52 ± 0.21 ^c^	
	330	78.54 ± 0.15 ^d^	
AgNPs	125	86.06 ± 0.39 ^b^	14.07
	250	83.38 ± 0.17 ^c^	
	500	82.52 ± 0.17 ^c^	
Acarbose	2.5	90.41 ± 0.22 ^e^	N/A

Data presented as mean ± SD (n = 3 independent experiments). Different superscript letters indicate significant differences between groups (*p* < 0.05, one-way ANOVA with Tukey’s post hoc test). CE, crude extract; AgNPs, silver nanoparticles. Note the non-monotonic dose–response relationship with maximal inhibition occurring at lowest tested concentrations. Different superscript letters (a, b, c, d, e) indicate statistically significant differences between groups (*p* < 0.05, one-way ANOVA with Tukey’s post hoc test). Groups sharing the same letter are not significantly different.

**Table 4:** α-Glucosidase Inhibitory Activity of *S. birrea* Formulations. Data are presented as mean ± SD from three independent experiments (n = 3), each performed with three technical replicates (total 9 measurements per condition). Different superscript letters (a, b, c, e) indicate statistically significant differences between groups (*p* < 0.05, one-way ANOVA with Tukey’s post hoc test for multiple comparisons). IC_50_ values were calculated by nonlinear regression analysis (log[concentration] vs. response, variable slope four-parameter model) using GraphPad Prism 8.0 software. CE, crude extract; AgNPs, silver nanoparticles.

Treatment Group	Concentration (µg/mL)	α-Glucosidase Inhibited (%)	IC_50_ (µg/mL)
Untreated	0	0 ± 0 ^e^	
CE	82	91.86 ± 0.42 ^a^	15.96
	164.4	88.05 ± 0.82 ^b^	
	330	83.60 ± 0.21 ^c^	
AgNPs	125	94.73 ± 0.70 ^a^	15.82
	250	90.57 ± 0.15 ^ab^	
	500	88.73 ± 0.29 ^b^	
Acarbose	2.5	94.48 ± 0.44 ^a^	N/A

Data are presented as mean ± SD from three independent experiments (n = 3), each performed with three technical replicates (total 9 measurements per condition). Different superscript letters (a, b, c, e) indicate statistically significant differences between groups (*p* < 0.05, one-way ANOVA with Tukey’s post hoc test for multiple comparisons). Groups sharing the same letter are not significantly different. IC_50_ values were calculated by nonlinear regression analysis (log[concentration] vs. response, variable slope four-parameter model) using GraphPad Prism 8.0 software. CE, crude extract; AgNPs, silver nanoparticles. Note the non-monotonic dose–response relationship with maximal inhibition at lowest tested concentrations. Different superscript letters (a, b, c, e) indicate statistically significant differences between groups (*p* < 0.05, one-way ANOVA with Tukey’s post hoc test). Groups sharing the same letter are not significantly different.

**Table 5:** DPP-IV Inhibitory Activity of *S. birrea* Formulations. Data are presented as mean ± SD from three independent experiments (n = 3), each performed with three technical replicates (total 9 measurements per condition). Different superscript letters (a, b, c, d, e) indicate statistically significant differences between groups (*p* < 0.05, one-way ANOVA with Tukey’s post hoc test for multiple comparisons). IC_50_ values were calculated by nonlinear regression analysis (log[concentration] vs. response, variable slope four-parameter model) using GraphPad Prism 8.0 software. CE, crude extract; AgNPs, silver nanoparticles.

Treatment Group	Concentration (µg/mL)	DPP-IV Inhibited (%)	IC_50_ (µg/mL)
Untreated	0	0 ± 0 ^a^	
CE	82	0 ± 0 ^a^	ND
	164.4	0 ± 0 ^a^	
	330	0 ± 0 ^a^	
AgNPs	125	31.06 ± 0.33 ^b^	220.5
	250	56.61 ± 0.15 ^c^	
	500	72.45 ± 0.18 ^d^	
Sitagliptin	1 µM	90.41 ± 0.22 ^e^	N/A

Data presented as mean ± SD (n = 3 independent experiments). Different superscript letters indicate significant differences between groups (*p* < 0.001, one-way ANOVA with Tukey’s post hoc test). Note: CE showed no detectable DPP-IV inhibition at any concentration tested (all values identical to untreated, *p* = 1.000). ND, not determined due to <10% inhibition. AgNPs demonstrated concentration-dependent inhibition with excellent linear correlation (R^2^ = 0.9987, slope = 0.109 ± 0.005). Different superscript letters (a, b, c, d, e) indicate statistically significant differences between groups (*p* < 0.05, one-way ANOVA with Tukey’s post hoc test). Groups sharing the same letter are not significantly different.

## Data Availability

The data presented in this study are available on request from the corresponding author. Raw data supporting the findings of this study are available from the corresponding author upon reasonable request due to ongoing patent applications related to the biosynthesized silver nanoparticles.

## Supplementary Materials

The following supporting information can be downloaded at: https://www.mdpi.com/article/10.3390/ijms27062584/s1. References [69,70,71,72,73] are cited in the Supplementary Materials.

## Abbreviations

The following abbreviations are used in this manuscript:

AgNPs	Silver nanoparticles
AMPK	AMP-activated protein kinase
ANOVA	Analysis of variance
ATP	Adenosine triphosphate
CE	Crude extract
CNP	2-Chloro-4-nitrophenol
CNPG3	2-Chloro-4-nitrophenol α-D-maltotrioside
DLS	Dynamic light scattering
DM	Diabetes mellitus
DMEM	Dulbecco’s Modified Eagle Medium
DMSO	Dimethyl sulfoxide
DPP-IV	Dipeptidyl peptidase-IV
DPPH	2.2-Diphenyl-1-picrylhydrazyl
FCC	Face-centered cubic
FCS	Fetal calf serum
FTIR	Fourier-transform infrared spectroscopy
FWHM	Full width at half maximum
GIP	Glucose-dependent insulinotropic polypeptide
GLP-1	Glucagon-like peptide-1
GLUT2	Glucose transporter 2
GOD-POD	Glucose oxidase-peroxidase
H_2_O_2_	Hydrogen peroxide
HbA1c	Glycated hemoglobin
IC_50_	Half-maximal inhibitory concentration
IC_10_	10% inhibitory concentration
LC-MS/MS	Liquid chromatography-tandem mass spectrometry
PDI	Polydispersity index
pNPG	p-Nitrophenyl-α-D-glucopyranoside
ROS	Reactive oxygen species
*S. birrea*	*Sclerocarya birrea*
SD	Standard deviation
SEM	Scanning electron microscopy
SGLT1	Sodium-dependent glucose transporter 1
SPR	Surface plasmon resonance
T2DM	Type 2 diabetes mellitus
TAC	Total antioxidant content
TEM	Transmission electron microscopy
XRD	X-ray diffraction
