# Supplementary material for: Anti-Hyperglycemic and Antioxidant Effects of Sclerocarya birrea Leaf Crude Extract and Biosynthesized Silver Nanoparticles In Vitro
Source: Int J Mol Sci. 2026 Mar 11;27(6):2584. doi: 10.3390/ijms27062584 (PMC13026587; doi:10.3390/ijms27062584)
Supplement: Supplementary file 1 [file ijms-27-02584-s001.zip › ijms-4119275-supplementary.pdf]

Supplementary materials

Characterization of Synthesized Silver Nanoparticles

UV-Vis Absorption Spectra Analysis

The formation of the AgNPs was confirmed by measuring the surface plasmon resonance (SPR) peak in the UV-Vis spectra. The UV-Vis spectra showed an intense SPR peak at around 451 nm. The shape, size, morphology, and composition of the synthesized silver nanoparticles directly influenced this SPR band.

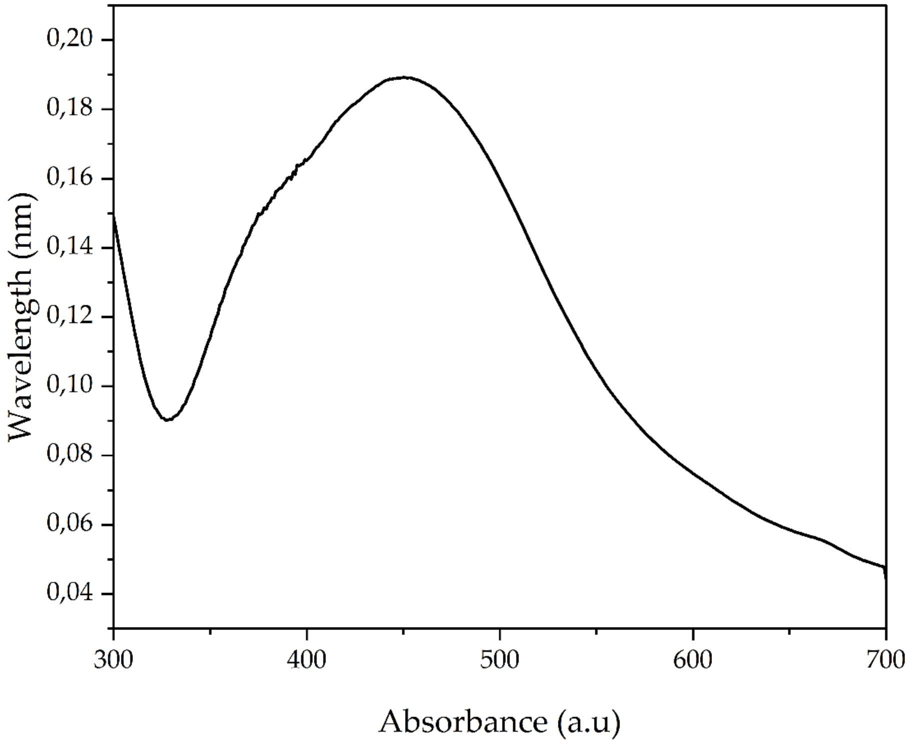

Figure S1. UV-Visible absorption spectra of *S. birrea* leaf AgNPs showing characteristic surface plasmon resonance peak at 451 nm, confirming successful nanoparticle synthesis

X-Ray Diffraction (XRD) Analysis

X-ray diffraction was used to investigate the crystalline structure and lattice characteristics of the synthesized *S. birrea* leaf AgNPs.

For the synthesised *S. birrea* leaf AgNPs, five sharp peaks were observed at 2θ values 37.93°, 44.08°, 64.10°, 76.97° and 81.07°. These 2θ values correspond to the diffraction planes (111), (200), (220), (311), and (222) of the face-centered cubic (FCC) structure of the silver

nanoparticles. The maximum intensity peak was found at the (220) plane, which is the ideal orientation of the structure. Extra peaks were observed, which are attributed to the biological components in the extract.

The crystallite size was calculated using the Debye-Scherrer formula.

Table S1: XRD analysis results for *S. birrea* leaf AgNPs showing 2θ values (37.93°, 44.08°, 64.10°, 76.97°, 81.07°), corresponding diffraction planes (111, 200, 220, 311, 222), FWHM (0.295 radians for the 111 plane), and calculated crystallite size (32.1 nm using Debye-Scherrer equation).

**Sample**	**Intense peak position (2θ)**	**Diffraction plane (hkl)**	**FWHM, (β) radians**	**Crystalline size (D), nm**	**JCPDS Ref**
S. birrea leaf AgNPs	44.08	200	0.295	32.1	04-0783

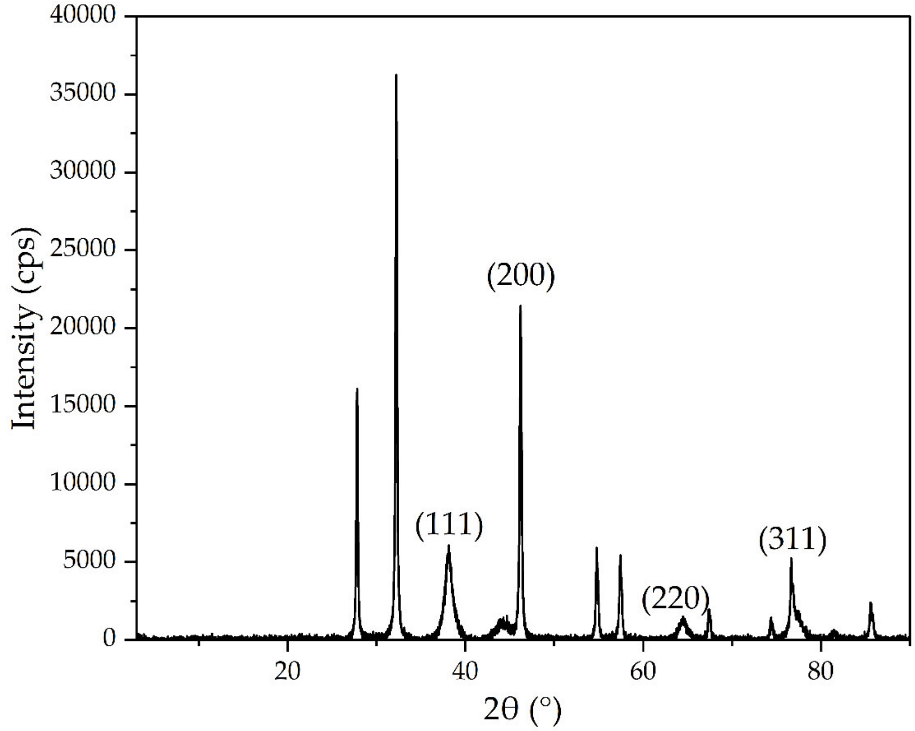

Figure S2. X-ray diffraction (XRD) pattern of *S. birrea* leaf AgNPs showing face-centered cubic (FCC) crystal structure with five characteristic diffraction peaks at 2θ = 37.93°, 44.08°, 64.10°, 76.97°, and 81.07°, corresponding to (111), (200), (220), (311), and (222) crystallographic planes. Additional minor peaks attributed to crystallized bioorganic components from plant extract

Transmission Electron Microscopy (TEM) Analysis

TEM was used to determine the size and morphology of the synthesized *S. birrea* leaf AgNPs.

The magnified TEM image of the *S. birrea* leaf AgNPs showed agglomeration of the silver nanoparticles. Most particles were spherical. The particle-size distribution ranged from 22–50 nm, with an average of 36.8 ± 8.6 nm as revealed by the histogram. This average size is noted to be close to the value obtained using the XRD method.

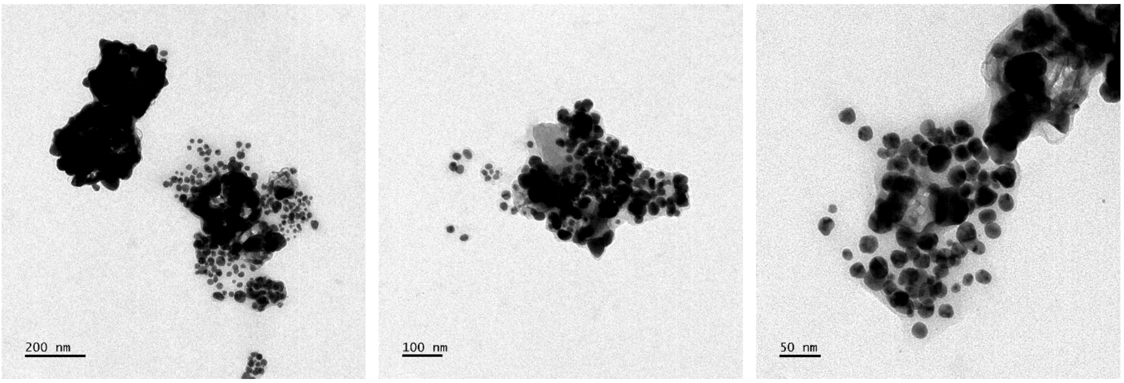

Figure S3a. Transmission electron microscopy (TEM) images of *S. birrea* leaf AgNPs at multiple magnifications showing predominantly spherical morphology with some agglomeration, and particle size distribution histogram derived from measurement of >100 particles (average size: 36.8 ± 8.6 nm, range: 22–50 nm).

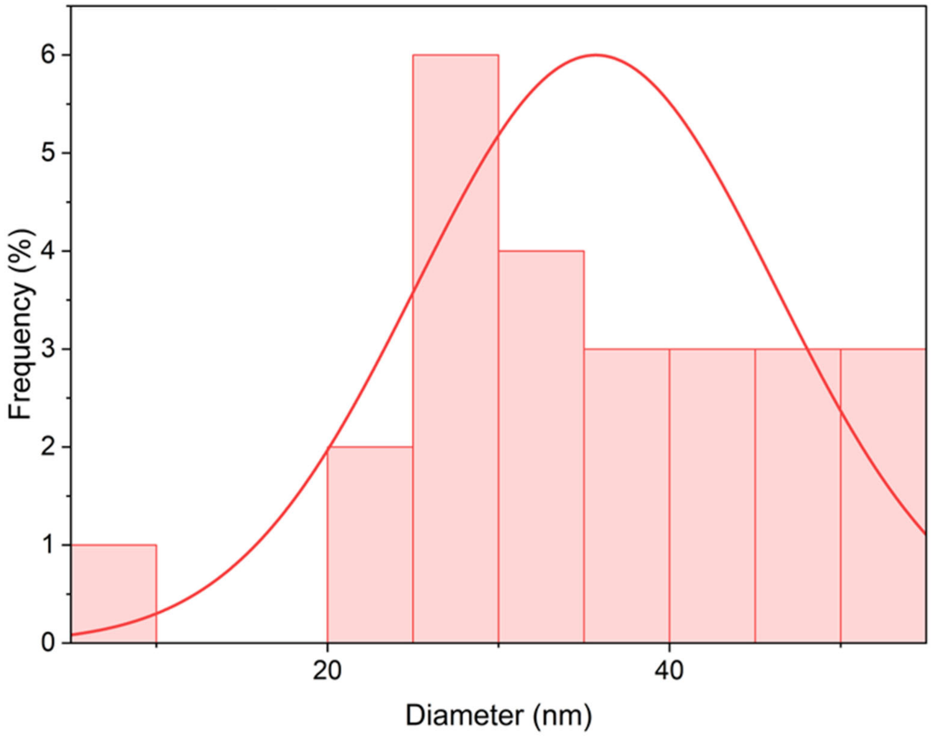

Figure S3b. The particle size distribution obtained from several TEM images for *S. birrea* leaf AgNPs

Scanning Electron Microscopy (SEM) Analysis

SEM was used to determine the size and morphology of the synthesized AgNPs. The magnified SEM image of the second batch of the silver nanoparticles showed **agglomeration** of the silver nanoparticles. Most particles were spherical in shape.

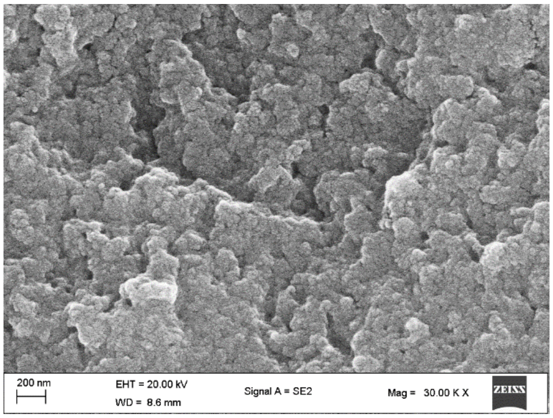

Figure S4. Scanning electron microscopy (SEM) image of S. birrea leaf AgNPs showing spherical particles with some degree of agglomeration, consistent with modest zeta potential

Dynamic Light Scattering (DLS)

DLS was used to evaluate the average particle size and polydispersity index (PDI) of the AgNPs in solution.

The average size of *S. birrea* leaf AgNPs was 220 nm (hydrodynamic diameter). The PDI was 0.334. Since the PDI value is less than 0.7, the silver nanoparticles synthesized with *S. birrea* leaves are considered moderately polydisperse (PDI = 0.334).

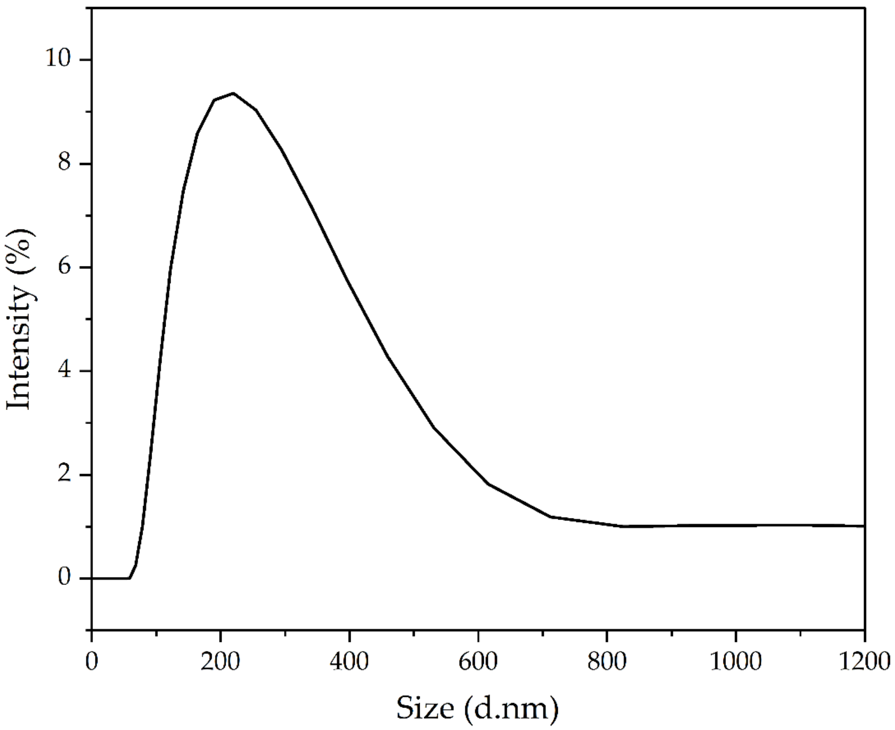

Figure S5. Dynamic light scattering (DLS) size distribution showing hydrodynamic diameter of 220 nm with polydispersity index (PDI) of 0.334, indicating moderately polydisperse distribution

Zeta Potential

The zeta potential value for the second batch of AgNPs was -19.5 mV ± 8.36 mV. The measurement gave a single peak with a peak-to-area ratio of 19.5 mV/100.0%.

The zeta potential of -19.5 mV indicates modest colloidal stability. While zeta (ζ) $\geq$ 30 mV typically denotes strong long-term stability, the measured value suggests moderate electrostatic repulsion, which explains the agglomeration observed in SEM images. However, the AgNPs prepared have a negative zeta potential value (−19.5 mV), indicating more electrostatic repulsion between the particles and modest stability; partial agglomeration observed. The negative zeta potential is likely due to the potential capping of bioorganic components present in the plant extract.

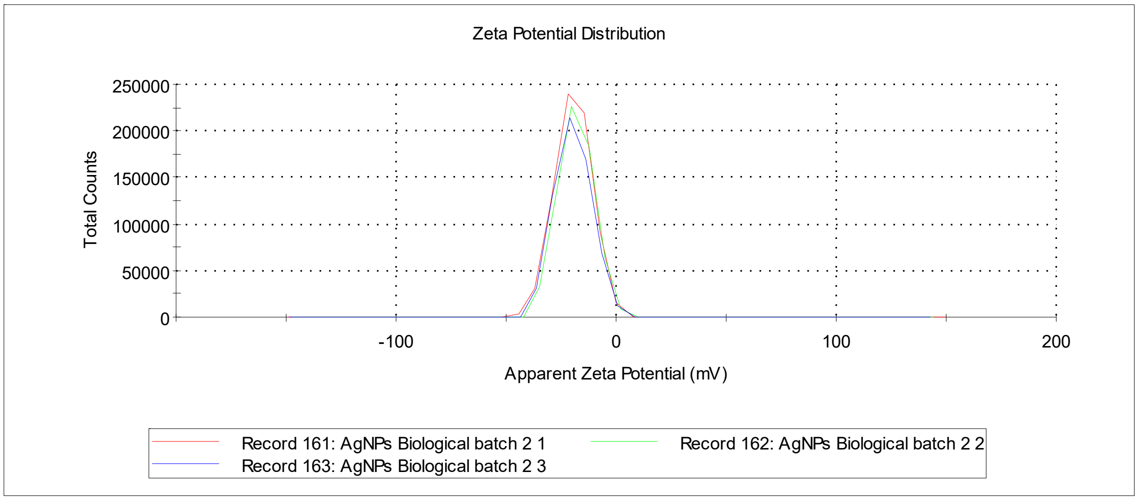

Figure S6. Zeta potential distribution of *S. birrea* leaf AgNPs showing peak at −19.5 mV ± 8.36 mV with peak-to-area ratio of 19.5 mV/100.0%, indicating modest colloidal stability

Fourier Transform Infra-Red (FTIR) Analysis

FTIR spectroscopy was used to identify the potential biomolecules responsible for the reduction and stabilization of the *S. birrea* leaf AgNPs.

The *S. birrea* leaf AgNPs synthesized from *S. birrea* leaf crude extract showed characteristic absorption peaks for various functional groups:

- OH (hydroxyl group): 3653.16 cm^{-1}
- N-H (amine group): 3223.91 cm^{-1}
- C-H (alkane group): 2916.88 cm^{-1}
- C=O (carboxyl group): 1604 cm^{-1}
- CO–O–CO (anhydride group): 1030.4 cm^{-1}
- Aromatic (bending vibrations): 873.92 cm^{-1}

The similarities between the peaks in the spectra of the plant extract and the nanoparticles, with some nominal shifts, confirmed the presence of the plant extract as capping and stabilizing agents. The presence of the C=O peak indicated that carbonyl-containing functional groups may be responsible for the bio-reduction of silver ions. The shifting of N-H stretching vibrations to lower wavelengths suggested the binding of these functional groups with the surface of the silver nanoparticles.

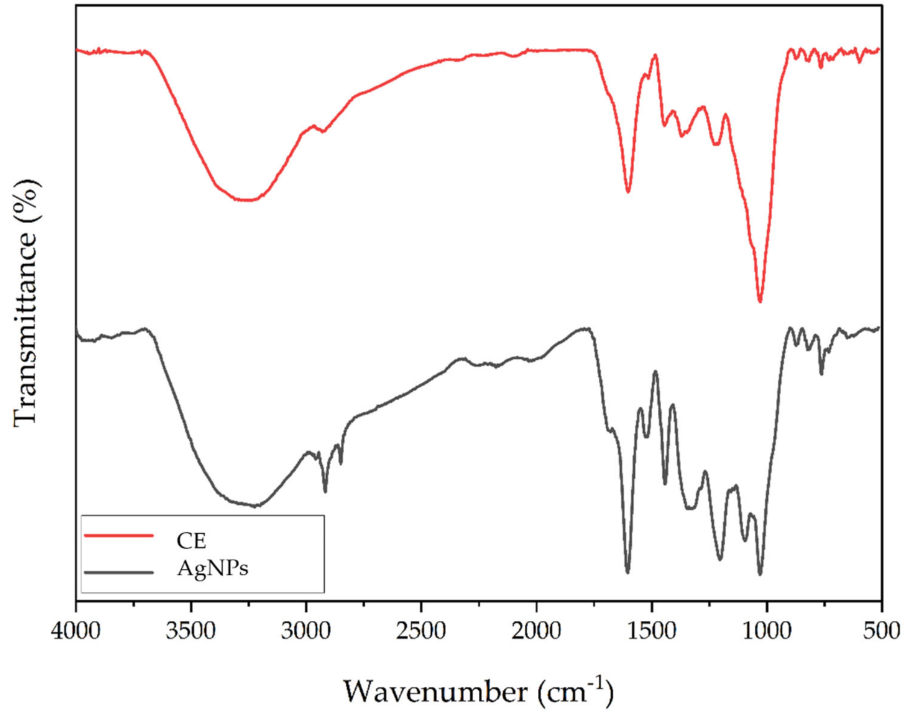

Figure S7. Fourier-transform infrared (FTIR) spectra comparing *S. birrea* leaf crude extract (CE) and AgNPs, showing functional groups involved in nanoparticle stabilization. Key peaks for AgNPs: 3653.16 cm^{-1} (O-H), 3223.91 cm^{-1} (N-H, shifted from 3244.21 cm^{-1} in CE), 2916.88 cm^{-1} (C-H), 1604 cm^{-1} (C=O), 1030.4 cm^{-1} (CO-O-CO), 873.92 cm^{-1} (aromatic).

Alpha amylase inhibition for IC50 correction

Table S2: Results for *S. birrea* leaf AgNPs showing alpha amylase inhibition using doses above the cut off points for IC50 verification.

CE (µg/mL)	Alpha amylase inhibited (%)	AgNPs (µg/mL)	Alpha amylase inhibited (%)
0	0.00	0	0.00
625	61.76	625	78.72
1250	38.73	1250	77.70
2500	-9.33	2500	66.57

CE (µg/mL)	Alpha amylase inhibited (%)	AgNPs (µg/mL)	Alpha amylase inhibited (%)
5000	-105.16	5000	53.15

Alpha glucosidase inhibition for IC50 correction

Table S3: Results for *S. birrea* leaf AgNPs showing alpha glucosidase inhibition using doses above the cut off points for IC50 verification.

CE (µg/mL)	Alpha glucosidase inhibited (%)	AgNPs (µg/mL)	Alpha glucosidase inhibited (%)
0	0.00	0	0.00
625	56.47	625	79.60
1250	26.65	1250	79.10
2500	-27.74	2500	68.26
5000	-130.42	5000	53.79

DPP IV inhibition by Silver Nitrate

Table S4: DPP-IV inhibition by $AgNO_3$ (ionic silver) across concentrations matching the AgNP assay range.

$AgNO_3$ (µg/mL)	DPP IV inhibited (%)
0	0.00
125	0.89

$AgNO_3$ (μg/mL)	DPP IV inhibited (%)
250	0.98
500	1.77

DPPH inhibition for IC50 correction

Table S5: DPPH radical scavenging activity of CE and AgNPs at extended concentrations (for curve-shape verification).

CE (μg/mL)	DPPH inhibited (%)	AgNps (μg/mL)	DPPH inhibited (%)
0	0.00	0	0.00
625	69.20	625	73.70
1250	62.82	1250	70.98
2500	50.28	2500	68.49
5000	28.31	5000	64.47

References

1. A. Aruna, R. Nandhini, V. Karthikeyan and P. Bose, *Asian journal of biomedical and pharmaceutical sciences*, 2014, **4**, 1.
2. T. J. I. Edison and M. Sethuraman, *Process Biochemistry*, 2012, **47**, 1351-1357.
3. D. Sivaraman, P. Panneerselvam, P. Muralidharan, T. P. Prabhu and R. V. Kumar, *International Journal of Pharmaceutical Sciences and Research*, 2013, **4**, 2280.
4. D. N. de Assis, V. C. F. Mosqueira, J. M. C. Vilela, M. S. Andrade and V. N. Cardoso, *International journal of pharmaceutics*, 2008, **349**, 152-160.
5. S. Honary, H. Barabadi, E. Gharaei-Fathabad and F. Naghibi, *Tropical Journal of Pharmaceutical Research*, 2013, **12**, 7-11.
